# Engineering a new-to-nature cascade for phosphate-dependent formate to formaldehyde conversion in vitro and in vivo

**DOI:** 10.1038/s41467-023-38072-w

**Published:** 2023-05-09

**Authors:** Maren Nattermann, Sebastian Wenk, Pascal Pfister, Hai He, Seung Hwan Lee, Witold Szymanski, Nils Guntermann, Fayin Zhu, Lennart Nickel, Charlotte Wallner, Jan Zarzycki, Nicole Paczia, Nina Gaißert, Giancarlo Franciò, Walter Leitner, Ramon Gonzalez, Tobias J. Erb

**Affiliations:** 1 Department of Biochemistry & Synthetic Metabolism, Max Planck Institute for Terrestrial Microbiology, Marburg, Germany; 2 Max Planck Institute of Molecular Plant Physiology, Potsdam, Germany; 3 Department of Chemical, Biological, and Materials Engineering, University of South Florida, Tampa, FL USA; 4 Institute of Translational Proteomics, Philipps University, Marburg, Germany; 5 Institute of Technical and Macromolecular Chemistry, RWTH Aachen University, Aachen, Germany; 6 Ruprecht Karl University, Heidelberg, Germany; 7 Philipps University, Marburg, Germany; 8 Core Facility for Metabolomics and Small Molecule Mass Spectrometry, Max Planck Institute for Terrestrial Microbiology, Marburg, Germany; 9 Festo SE & Co. KG, Esslingen, Germany; 10 Max Planck Institute for Chemical Energy Conversion, Mülheim an der Ruhr, Germany; 11 Center for Synthetic Microbiology (SYNMIKRO), Marburg, Germany

**Keywords:** Biocatalysis, Metabolic engineering, Synthetic biology, Applied microbiology

## Abstract

Formate can be envisioned at the core of a carbon-neutral bioeconomy, where it is produced from CO_2_ by (electro-)chemical means and converted into value-added products by enzymatic cascades or engineered microbes. A key step in expanding synthetic formate assimilation is its thermodynamically challenging reduction to formaldehyde. Here, we develop a two-enzyme route in which formate is activated to formyl phosphate and subsequently reduced to formaldehyde. Exploiting the promiscuity of acetate kinase and *N-*acetyl-γ-glutamyl phosphate reductase, we demonstrate this phosphate (P_i_)-based route in vitro and in vivo. We further engineer a formyl phosphate reductase variant with improved formyl phosphate conversion in vivo by suppressing cross-talk with native metabolism and interface the P_i_ route with a recently developed formaldehyde assimilation pathway to enable C2 compound formation from formate as the sole carbon source in *Escherichia coli*. The P_i_ route therefore offers a potent tool in expanding the landscape of synthetic formate assimilation.

## Abstract (web summary)

A key step in the assimilation of formate is its reduction into formaldehyde. Here, the authors develop a two-enzyme route in which formate is activated into formyl phosphate and reduced by NAD(P)H into formaldehyde and confirm its functionality in vitro and in vivo.

## Introduction

One of the major steps towards establishing a carbon-neutral (or carbon-positive) bio-economy is the valorization of carbon dioxide as a feedstock. While a variety of synthetic CO_2_-fixing cycles and microorganisms have been described recently^1–3^, the direct use of CO_2_ in biotechnology still poses several challenges and limitations. An alternative is the chemical conversion of CO_2_ into soluble one-carbon (C1) compounds that can be further converted by biological platform organisms. One of the most promising C1-molecules in such chemo-bio hybrid approaches is formate. Formate can be produced electrochemically or by catalytic hydrogenation^4–6^, is fully miscible with water, non*-*flammable and of little toxicity to the human worker^7^.

**Fig. 1:**
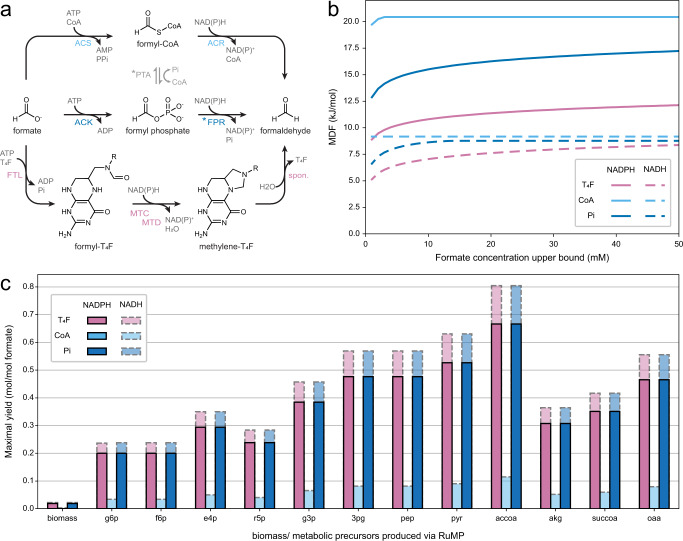
Design and analysis of different pathways for the reduction of formate to formaldehyde. **a** Reactions schemes for the different formate reduction routes discussed in this study. The enzymes for each respective route are provided using the following color-coding: T_4_F route (purple), CoA route (light blue), Pi route (dark blue). PTA, which can be used to toggle between CoA and Pi routes, is shown in gray. * indicates (plausible) new-to-nature reactions. ACS acetyl-CoA synthetase, ACR acyl-CoA reductase, PTA phosphate acetyl transferase, ACK acetate kinase, FPR formyl phosphate reductase, FTL formate tetrahydrofolate ligase, MTC methylene tetrahydrofolate cyclohydrolase, MTD methylene tetrahydrofolate dehydrogenase, T_4_F tetrahydrofolate, spon. spontaneous hydrolysis. **b** MDF calculations for the three routes with either NADPH (solid line) or NADH (dashed line) as reduction equivalent. Energy profiles of individual reactions are given in Supplementary Table 1. **c** FBA calculations for the production of 12 relevant metabolic precursors as well as biomass via the three routes and the RuMP pathway in *E. coli*. g6p glucose 6-phosphate, f6p fructose 6-phosphate, e4p erythrose 4-phosphate, r5p ribose 5-phosphate, g3p glyceraldehyde 3-phosphate, 3pg glycerate 3-phosphate, pep phosphoenolpyruvate, pyr pyruvate, accoa: acetyl-CoA, akg 2-ketoglutarate, succoa: succinyl-CoA, oaa oxaloacetate. Source data are provided as a Source Data file.

To develop a formate-based bioeconomy, recent efforts have focused on converting common platform organisms, such as *Escherichia coli* into synthetic formatotrophs that accept formate as both carbon and energy source^8,9^. Formatotrophy naturally relies on the activation of formate to the tetrahydrofolate (T_4_F) cofactor and subsequent reduction to methylene-T_4_F (T_4_F cascade, Fig. 1). Methylene-T_4_F can be channeled into central metabolism directly via the serine cycle^10^ or the reductive glycine pathway^11^. Alternatively, methylene-T_4_F can spontaneously hydrolyze, releasing formaldehyde, which can enter central carbon metabolism through the ribulose monophosphate pathway (RuMP)^12^, xylulose monophosphate pathway^13^, homoserine cycle^14^, or formolase pathways^15–19^, although the spontaneous hydrolysis rate is too slow to fully sustain growth. An alternative to growth-coupled metabolic integration are orthogonal systems for value-added product generation, such as the FORCE pathway^20^.

To overcome the limitations of the natural methylene-T_4_F route, different synthetic pathways have been proposed and/or developed. One example is the activation of formate to formyl-CoA, followed by the reduction to formaldehyde through the promiscuous activities of acetyl-CoA synthetase (ACS) and acyl-CoA reductase (ACR)^15, 19,21,22^. Compared to the natural T_4_F cascade, no spontaneous hydrolysis is required and one less enzyme is needed. However, the CoA-based cascade requires hydrolysis of one additional phosphoanhydride bond for activation (ATP → AMP + PP_i_, Fig. 1a). While the CoA-based route has been demonstrated in a proof-of-principle, residual activities of the synthetase with acetate and ACR with the native CoA metabolite pool (e.g., acetyl-CoA) currently limit the application of the CoA-based route in vivo^19^.

Another way to activate carboxylic groups prior to reduction is through phosphorylation. Recently, a three-enzyme route was proposed, in which formate is first activated to formyl phosphate (using acetate kinase, ACK), transferred to CoA via phosphate and further reduced by acyl-CoA reductase^20,23,24^. However, this route still relies on an acyl-CoA reductase and thus faces the same interference with the acetyl-CoA pool. To circumvent this challenge, the direct reduction of formyl phosphate would be a highly attractive alternative.

Here, we seek to establish the direct reduction of formyl phosphate into formaldehyde through developing a new-to-nature enzyme, formyl phosphate reductase (FPR). We identify promiscuous FPR activity in four enzyme families and present one homolog of *N-*acetyl-γ-glutamyl phosphate (NAGP) reductase from *Denitrovibrio acetiphilus* (DaArgC) that produces relevant levels of formaldehyde. Using a semi-rational enzyme engineering approach, we create a triple substitution variant of the enzyme (DaArgC3) that shows a complete loss of the native enzyme activity (i.e., NAGP reduction), a 300-fold shift in specificity towards formyl phosphate, and a decreased side reactivity with acetyl phosphate. Combining DaArgC3 with ACK allows us to establish a route to formaldehyde that is completely based on phosphate (P_i_). This P_i_-based route outcompetes synthetic CoA-based routes in respect to thermodynamic efficiency and T_4_F-based routes in respect to kinetic considerations. Interfacing our P_i_-based route with the FORCE pathway enables the production of C2 compounds in vitro and in vivo from formate as the sole carbon source, underlining the biotechnological potential of the P_i_ route.

## Results

### Design and evaluation of a P_i_-based route to formaldehyde

While different formaldehyde forming pathways from an activated formyl-group had been suggested in literature^15,18–20^, to the best of our knowledge, a reduction of a formyl phosphate had not been considered so far. Expanding the solution space by this hypothetical reaction allowed us to draft a completely P_i_-based route (Fig. 1a). To understand how this new-to-nature route compares to its alternatives (i.e., the synthetic CoA-based and the natural T_4_F-dependent routes; Fig. 1a), we evaluated the thermodynamic profiles of the three different routes with Max-min Driving Force (MDF) calculations (Fig. 1b). All routes show a positive driving force (indicating thermodynamic feasibility) that was higher when NADPH was used instead of NADH, due to the more favorable intracellular NAD(P)H:NA(D)P^+^ ratios^25^. While the CoA-based route had the highest max–min driving force, flux balance analysis (FBA) showed that the expected biomass yield of the RuMP cycle through the CoA-based route was significantly lower compared to the biomass yield of the RuMP cycle through the T_4_F- and the P_i_-based route, respectively (Fig. 1c). Overall, these calculations confirmed that our P_i_-based route outcompetes the synthetic CoA-based route in respect to thermodynamic considerations, notably, without relying on a spontaneous hydrolysis reaction like in the natural T_4_F-based route, thus being kinetically favored in respect to the latter.

### Screening acetate kinases for formate kinase activity

To establish the P_i_-based route, we first focused on identifying a formate kinase (FOK) that could catalyze the phosphorylation of formate. Only very few examples of FOK activity have been reported, however, no corresponding genes have been identified, thus far^23^. Nevertheless, an acetate kinase from *Salmonella typhimurium* (StAckA) was described to catalyze the phosphorylation of formate^24^. We characterized the FOK activity of StAckA and its *E. coli* homolog (EcAckA) in more detail. Based on its slightly more favorable catalytic properties, we chose EcAckA for our formate reduction cascade (Table 1 and Supplementary Fig. 1).

**Table 1:** Kinetic parameters of acetate kinases on formate

	*K*_m, app_ (mM)	*k*_cat, app_ (s^−1^)	*k*_cat_/*K*_m_ (M^−1^s^−1^)
EcAckA	106 ± 10	22 ± 0.9	2.1 × 10^2^
StAckA	117 ± 9	16 ± 0.5	1.4 × 10^2^

Shown are the results of a Michaelis-Menten fit of technical triplicates (*n* = 3) with standard deviation. Curves are provided in Supplementary Fig. 1.

### Screening identifies FPR activity in different enzymes

**Fig. 2:**
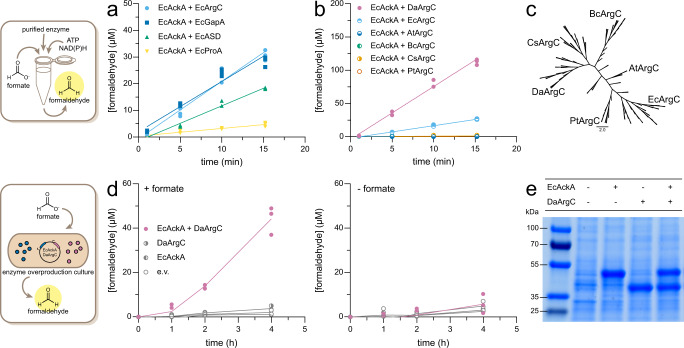
Discovery of promiscuous formyl phosphate reductase activity in diverse metabolic enzymes. **a** Comparison of multiple *E. coli* enzymes’ formyl phosphate reductase activity. Formyl phosphate was produced in situ by EcAckA (from 50 mM NH_4_ formate). Formaldehyde was detected by Nash assay. Individual measurements and linear regression of the mean are shown. Data points represent independent technical replicates (*n* = 3). Data was baseline-corrected against a no formate control. Negative controls are depicted in Supplementary Fig. 2. **b** Comparison of ArgC homologs regarding their activity toward formyl phosphate. Assay was performed as in **a**. Individual measurements and linear regression of the mean are shown. Data points represent independent technical replicates (*n* = 3). Negative controls are depicted in Supplementary Fig. 3. **c** Phylogeny of ArgC homologs. 50x bootstrap Neighbor-Joining tree of homologs with <60% identity to EcArgC is shown. Chosen homologs are indicated on the tree. **d** Formaldehyde production by EcAckA + DaArgC in *E. coli* BL21 (DE3) Δ*frmRAB* in the presence or absence of 50 mM NH_4_ formate. Individual measurements and linear regression of the mean are shown. Data points represent independent biological replicates (*n* = 3). Formaldehyde was detected as in (**a**). **e** SDS-PAGE of EcAckA and DaArgC production in *E. coli* BL21 (DE3) Δ*frmRAB*. The gel is a representative image for independent biological replicates yielding the same result (*n* = 3). EcAckA *Escherichia coli* acetate kinase, AtArgC *Arabidopsis thaliana N-*acetyl-γ-glutamyl phosphate dehydrogenase, BcArgC *Bacillus clausii N-*acetyl-γ-glutamyl phosphate dehydrogenase, CsArgC *Caldicellulosiruptor saccharolyticus N-*acetyl-γ-glutamyl phosphate dehydrogenase, DaArgC *Denitrovibrio acetiphilus N-*acetyl-γ-glutamyl phosphate dehydrogenase, EcArgC *Escherichia coli N-*acetyl-γ-glutamyl phosphate dehydrogenase, PtArgC *Pseudidiomarina taiwanensis N-*acetyl-γ-glutamyl phosphate dehydrogenase.

Next, we focused on identifying a formyl phosphate reductase (FPR). While the specific reduction of formyl phosphate into formaldehyde does not occur in nature, the reduction of different acyl-phosphates into aldehydes does. We evaluated four different reductases that catalyze such reactions for activity towards formyl phosphate: NAGP reductase (ArgC), aspartate semialdehyde dehydrogenase (ASD), glyceraldehyde-3-phosphate dehydrogenase (GapA) and γ-glutamyl phosphate reductase (ProA) (for their native reactions, see Supplementary Fig. 2). Because formyl phosphate is unstable and difficult to isolate, we produced it in situ using EcAckA. When testing the four candidates from *E. coli* (EcASD, EcArgC, EcGapA, EcProA), we detected FPR activity in all cases, albeit at very low rates (Fig. 2a). EcArgC and EcGapA performed best under the chosen conditions, with formaldehyde production rates of 2.2 µM/min and 1.9 µM/min, respectively. Because EcGapA is a glycolytic enzyme and at the core of central carbon metabolism, we chose EcArgC as candidate, hypothesizing that it would interfere less with endogenous metabolism.

To screen for catalytic variability within the ArgC family, we constructed a phylogenetic tree of 118 ArgC homologs and selected five representative variants from distinct clades for further biochemical characterization (Fig. 2b, c). While four of the homologs displayed no FPR activity, ArgC from *D. acetiphilus* (DaArgC) showed a formaldehyde production rate of 8 µM/min, which was about fourfold higher compared to the P_i_ route with EcArgC as FPR (Fig. 2b). Notably, the rate of the EcAckA - DaArgC route compared very favorably to published cascades based on formyl-CoA as intermediate (5 µM/min in vitro, Supplementary Table 2). Hence, we aimed at implementing the EcAckA and DaArgC-based P_i_ route in vivo.

### Establishing the P_i_-based route in vivo

To test and implement the P_i_-based route in vivo, we expressed EcAckA and DaArgC from a pCDFDuet-1 vector in *E. coli* strain BL21 DE3 Δ*frmRAB*. This strain lacks the glutathione-dependent formaldehyde detoxification operon (*frmRAB*) to improve formaldehyde production. When adding 50 mM formate to a growing *E. coli* BL21 (DE3) Δ*frmRAB* pCDFDuet-1_EcAckA_DaArgC culture, we observed formaldehyde production at ~10 µM/h. Formaldehyde production was dependent on the expression of both enzymes (Fig. 2d), which were produced at approximately equal amounts (Fig. 2e, Supplementary Fig. 4). Formaldehyde production in the control without additional formate feeding was marginal and likely caused by basal formate levels produced in vivo by endogenous pyruvate-formate lyase (PFL), see below (Fig. 2d, Supplementary Fig. 5). To exclude any toxic effects of our assay setup onto *E. coli*, which could have affected formaldehyde production rates, we performed survival assays with our strains (Supplementary Fig. 6) and confirmed that all cells, including the controls (empty vector, DaArgC only, EcAckA only) could be successfully rescued in LB medium after exposure to the assay (Supplementary Fig. 7).

Based on the kinetic parameters of EcAckA and DaArgC (Tables 1 and 2), we speculated that DaArgC was the rate-limiting step in the cascade. To confirm this hypothesis, we prepared cell extracts, which we spiked with either purified DaArgC or EcArgC. These experiments indeed showed that the cascade was limited by formyl phosphate reduction (Supplementary Fig. 4), which was in line with the observed behavior of the purified enzymes. Overall, this data demonstrated that the P_i_ route was active in vivo, but still limited by FPR activity.

**Table 2:** Kinetic parameters of the DaArgC variants

Enzyme	Formyl phosphate				NADPH^a^				NADH^a^				NADPH/NADH
	*K*_m, app_ (mM)	*k*_cat, app_ (s^−1^)		*k*_cat_/*K*_m_ (M^−1^ s^−1^)	*K*_m, app_ (mM)	*k*_cat, app_ (s^−1^)		*k*_cat_/*K*_m_(M^−1^ s^−1^)	*K*_m, app_ (mM)	*k*_cat, app_ (s^−1^)		*k*_cat_/*K*_m_(M^−1^ s^−1^)	*k*_cat_/*K*_m (NADPH)_/*k*_cat_/*K*_m (NADH)_
DaArgC	No fit		0.12 ± 0.04^b^	-	0.16 ± 0.02		0.66 ± 0.03	4.1 × 10^3^	>500^c^		<0.005^c^	<1^c^	>4.1 × 10^3^
DaArgC1	31 ± 7		0.11 ± 0.01	2.4	0.04 ± 0.01		0.14 ± 0.02	3.5 × 10^3^	0.28 ± 0.05		0.08 ± 0.00	2.8 × 10^2^	1.3 × 10^1^
DaArgC2	38 ± 9		0.17 ± 0.02	3.1	0.06 ± 0.02		0.26 ± 0.03	4.3 × 10^3^	0.30 ± 0.04		0.12 ± 0.00	4.0 × 10^2^	1.1 × 10^1^
DaArgC3	18 ± 7		0.12 ± 0.02	4.6	0.05 ± 0.01		0.23 ± 0.02	4.6 × 10^3^	0.34 ± 0.05		0.09 ± 0.00	2.6 × 10^2^	1.8 × 10^1^

Michaelis-Menten fit and standard deviation of independent technical replicates (*n* = 3).

^a^Measurements coupled to EcAckA.

^b^Maximal activity observed, [formyl phosphate] = 46 mM.

^c^Activity below detection limit. Curves are provided in Supplementary Fig. 17.

### Engineering DaArgC towards improved FPR activity

**Fig. 3:**
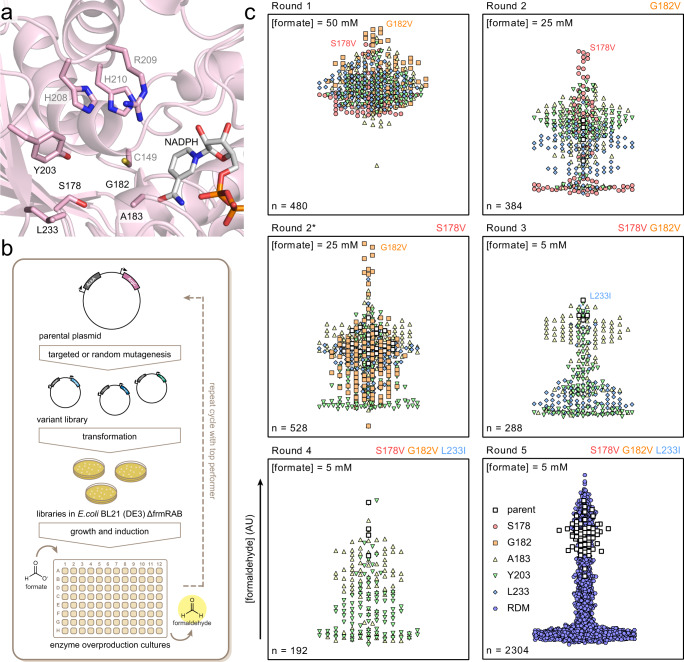
Enzyme engineering of DaArgC. **a** Cartoon depiction of the active site and substrate-binding domain of DaArgC (PBD-ID 8AFU, resolution 2.0 Å). NADPH (gray) is modeled based on the structure of MtArgC (PBD-ID 2I3G). The HRH motif responsible for binding of the phosphate and carbonyl group of the native substrate and the cysteine forming a covalent intermediate are labeled in light gray. Residues accommodating the branched side-chain of the native substrate are labeled in black. **b** Schematic depiction of the library screening method. **c** Iterative Saturation and Random Mutagenesis of DaArgC. Variants of DaArgC were generated by Saturation Mutagenesis (Rounds 1–4) or Random Mutagenesis (RDM) and produced in BL21 DE3 Δ*frmRAB*. Formate was added to the supernatant of the cultures as indicated on the top left of the graphs. After incubation, samples were taken and formaldehyde was detected by Nash assay to evaluate relative performance. Mutants displaying increased activity were used as parent in the next round as indicated above the graphs. Symbols represent individual measurements. EcAckA *Escherichia coli* acetate kinase, DaArgC *Denitrovibrio acetiphilus N-*acetyl-γ-glutamyl phosphate dehydrogenase, MtArgC *Mycobacterium tuberculosis N-*acetyl-γ-glutamyl phosphate dehydrogenase. Source data are provided as a Source Data file.

Because in vivo formaldehyde production was limited by DaArgC, we next aimed at improving the enzyme’s FPR activity through protein engineering. To that end, we solved the enzyme’s crystal structure and identified key active site residues for site-directed mutagenesis (Fig. 3a and Supplementary Fig. 8).

The ArgC active site can be divided into three parts, the NADPH-binding domain, the catalytic site, and a substrate-binding channel. The catalytic site consists of catalytically active C149 and the HRH motif (residues 208-210) responsible for binding the phosphate and carbonyl group of the substrate (gray labeling in Fig. 3a). The substrate channel includes a GAG motif (residues 182-184) and surrounding residues that accommodate the branched side-chain of *N-*acetyl-γ-glutamyl phosphate (NAGP) (black labeling in Fig. 3a).

To increase DaArgC activity with formyl phosphate, we aimed at improving accommodation of formyl phosphate in the active site by introducing larger amino acid residues in key positions to close up the active site of the enzyme, excluding binding of the native substrate. We targeted five residues of DaArgC (S178, G182, A183, Y203 and L233) (Fig. 3a) and used iterative saturation mutagenesis (ISM)^26^ to screen for favorable amino acid substitutions. ISM was performed in BL21 DE3 Δ*frmRAB* transformed with variants of pCDFDuet-1_EcAckA_DaArgC. Individual colonies were picked and subjected to in vivo screens in 96-well plates for formaldehyde production using the Nash assay (Fig. 3b). We screened approximately 4000 variants over 6 rounds of mutagenesis and incrementally lowered the formate concentration from 50 to 5 mM to screen for DaArgC variants with improved *K*_m_. Round 1 of mutagenesis resulted in variant DaArgC G182V (DaArgC1), which was followed by an S178V substitution in round 2 of mutagenesis (DaArgC2). Using double variant DaArgC2 as a template, we discovered a L233I substitution that showed an additional beneficial effect, resulting in triple variant DaArgC3. Although improvements were rather small, they were consistently discovered in replicates (Fig. 3c). When using DaArgC3 as template for iterative or random mutagenesis, no further improvements were discovered: round 4 yielded no further variants and round 5 only silent mutations.

### Validation of DaArgC variants in vivo and in vitro

**Fig. 4:**
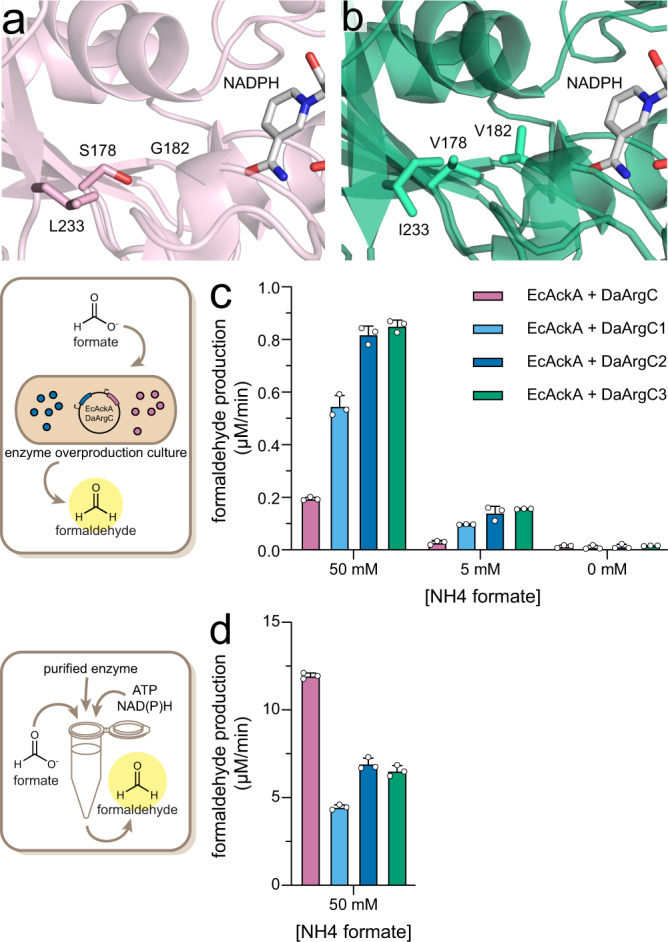
Validation of the DaArgC variants. **a** Crystal structure of DaArgC (PBD-ID 8AFU, 2.0 Å) and **b** DaArgC3 (PBD-ID 8AFV, 2.2 Å). NADPH (gray) is modeled based on the structure of *Mycobacterium tuberculosis* ArgC (PBD-ID 2I3G). Active site residues affected by mutagenesis are shown as sticks. **c** In vivo productivity of the DaArgC variants. EcAckA and DaArgC variants were produced in *E. coli* BL21 DE3 Δ*frmRAB* Δ*pflAB* and formate was added to the supernatant. The rate of formaldehyde production was evaluated by linear regression of four time points. Mean with standard deviation and individual data points are shown. Data points represent independent biological replicates (*n* = 3). **d** In vitro formaldehyde production of EcAckA + DaArgC variants. Data was evaluated as in **c**. Mean with SD and individual data points are shown. Data points represent independent technical replicates (*n* = 3). EcAckA *Escherichia coli* acetate kinase, DaArgC *Denitrovibrio acetiphilus N-*acetyl-γ-glutamyl phosphate dehydrogenase. Line plots of **c** and **d** are shown in Supplementary Fig. 9. Source data are provided as a Source Data file.

To validate the screened candidates, we expressed DaArgC WT and the three variants in *E. coli* BL21 DE3 Δ*frmRAB* Δ*pflAB*. Deletion of *pflAB* was used to suppress PFL-mediated background formate production in BL21 DE3 Δ*frmRAB*, which had caused significant variations in the negative control and at low formate feeding conditions (i.e., 5 mM formate, see Supplementary Fig. 5). When testing the different enzyme variants, in vivo, formaldehyde production increased from DaArgC to DaArgC3, while enzyme production levels remained unchanged, as judged by SDS-PAGE and LC-MS titer analysis (Fig. 4c, Supplementary Fig. 10). Across all substrate concentrations, DaArgC3 showed a fourfold improved formaldehyde productivity over wild-type (WT) DaArgC in vivo. Much to our surprise, however, in vitro activity of the variants had dropped between threefold (DaArgC1) and twofold (DaArgC2-3) compared to WT DaArgC (Fig. 4d). This finding raised the interesting question how mutagenesis had affected the kinetic parameter of the enzymes.

### DaArgC variants show decreased *K*_m_ for formyl phosphate

To understand why the engineered enzymes showed improved performance in in vivo assays, but not in vitro, we decided to characterize the different variants in more detail. To obtain substrate for assays, we chemically synthesized formyl phosphate from acetic formic anhydride and dipotassium phosphate. However, as formyl phosphate hydrolyzes in water over time (Supplementary Figs. 13 and 16), it was not possible to prepare standard solutions. Therefore, we synthesized formyl phosphate immediately added the crude product solution to the individual enzyme assay. This limited the maximum substrate concentration in our reaction mixtures to 50 mM, but allowed us to perform kinetic measurements over the first minutes, as the hydrolysis rate was negligible under these conditions (−0.34% min^−1^, Supplementary Fig. 16) compared to the enzymatic reaction. At 50 mM formyl phosphate, WT DaArgC showed an apparent *k*_cat_ of 0.1 s^−1^, but was not fully saturated. Variants DaArgC1, DaArgC2 and DaArgC3 showed similar turnover numbers (0.1–0.2 s^−1^), but were already saturated at apparent *K*_m_ (formyl phosphate) values around 20-30 mM, indicating that they performed better at formyl phosphate concentrations that are physiologically more relevant in vivo (Table 2, Supplementary Fig. 17).

### DaArgC variants show relaxed NAD(P)H specificity

We next coupled DaArgC variants to EcAckA to determine the kinetic parameters for the NADPH cofactor at constant production of formyl phosphate. WT DaArgC showed an apparent *K*_m_ (NADPH) of ~0.2 mM (at a *k*_cat_ of ~0.7 s^−1^), while variants DaArgC1-3 showed a fourfold decreased apparent *K*_m_ (NADPH) of ~0.05 mM (at *k*_cat_ between 0.2 and 0.3 s^−1^). Surprisingly, when testing NADH as alternative cofactor, which is not accepted by WT DaArgC, we found that DaArgC1-3 could use NADH at about 10% catalytic efficiency compared to NADPH (Table 2). The apparent *K*_m_ for NADH (~0.3 mM) was approximately sixfold higher compared to NADPH, while the specific activities were at about 50% of the activity with NADPH (~0.1 s^−1^), indicating that variants DaArgC1-C3 had evolved to also accept NADH as redox cofactor, which they could additionally access in vivo besides NADPH.

We thus aimed to understand the molecular basis for the relaxed cofactor selectivity in DaArgC1-3. When we re-established the glycine residue at position 182 in DaArgC2 and DaArgC3 (creating variants DaArgC2* and DaArgC3*), NADPH selectivity of the variants was fully restored, indicating that the glycine to valine substitution was responsible for the observed cofactor flexibility (Supplementary Table 3, Supplementary Fig. 17). This dramatic influence of a single residue on cofactor specificity came much to our surprise, as changes from NADPH to NADH selectivity usually require more intense engineering efforts^27,28^.

Solving the crystal structure of DaArgC3 suggested that the position of residues T9, S33, E34 and T35 (numbering according to mtArgC^29^) that interact with the 2′ phosphate of NADPH was not significantly affected by the G182V mutation (Supplementary Fig. 18). However, we noted that G182 lies within the conserved GAG motif of the substrate-binding channel (residues 182–184) and is flanked by two amino acids, T181 and R185, that form hydrogen bonds to the nicotinamide group and the diphosphate of NADPH, respectively (Supplementary Fig. 18). While we could not observed major changes between the TGAGR loop of DaArgC and the TVAGR loop of DaArgC3 in the NADP^+^/NADPH-unbound state (RMSD 0.119), it is known that in mtArgC^29^ the GAG motif moves closer to NADP^+^ in the bound state. This loop movement could be affected by the G182V substitution, resulting in a decreased *K*_m, app_ for NADPH (Table 2) and a relaxed cofactor specificity of the DaArgC1-3 mutants, providing a possible explanation for the observed changes in NAD(P)H binding.

### DaArgC variants exclude the native substrate NAGP

To further understand how the engineered enzymes had evolved, we determined the activity of WT and DaArgC variants towards their original substrate NAGP (Table 3). We produced NAGP in situ from *N-*acetyl-γ glutamate (NAG) and ATP, using *E. coli* acetyl glutamate kinase (ArgB). WT DaArgC showed a turnover of about 1 s^−1^ on NAGP, which is in line with activities of other ArgC homologs^30,31^. Notably, in DaArgC1-3, NAGP activity was reduced by almost three orders of magnitude compared to WT, indicating that the native activity was completely lost in these variants. Moreover, allosteric inhibition by NAG, the precursor of NAGP, was reduced fourfold in all variants compared to the WT (Table 3).

**Table 3:** Activity of the DaArgC variants on 50 mM *N-*acetyl-γ-glutamyl phosphate and acetyl phosphate and inhibition by *N-*acetyl-γ-glutamate

Enzyme	*N-*acetyl-γ-glutamyl phosphate		Acetyl phosphate		*N-*acetyl-γ-glutamate
	Turnover (s^−1^)	% activity	Turnover (s^−1^)	% activity	IC_50_ (mM)
DaArgC	0.903 ± 0.160	100	0.024 ± 0.001	100	5.1 ± 1.0
DaArgC1	0.002 ± 0.000	0.2	0.014 ± 0.001	58	17.7 ± 4.1
DaArgC2	0.002 ± 0.001	0.2	0.008 ± 0.000	33	24.4 ± 7.2
DaArgC3	0.001 ± 0.001	0.1	0.006 ± 0.000	25	24.6 ± 7.9

Mean and standard deviation of individual technical replicates (*n* = 3) are shown. Source data is provided in Supplementary Fig. 19.

Finally, we also tested for activity with acetyl phosphate, a native metabolite of *E. coli* that might act as competing substrate to formyl phosphate in vivo, especially when considering the strong overexpression of EcAckA in our engineered strains, which shows substantial acetyl-phosphate forming activity. Interestingly, acetyl phosphate reducing activity decreased fourfold from DaArgC to DaArgC3 (Table 3), indicating that this potentially competing side reaction became negligible in vivo.

Together, our results showed that during the course of engineering, variants DaArgC1-3 completely lost their native activity with NAGP without compromising FPR activity, which caused a switch in formyl phosphate selectivity between 50 and 300-fold. Moreover, these variants did no longer interfere with amino acid biosynthesis, were relieved of allosteric control by NAG and also show reduced side activity with acetyl phosphate, thus limiting interference with the acetate/acetyl-CoA pool, explaining why these variants overall performed better in vivo compared to the WT.

### Interfacing the P_i_-based route with the FORCE pathway

Having established and optimized the P_i_-based route to formaldehyde, we sought to further test this solution in combination with other downstream modules to build up more complex molecules from formate. Recently, the FORCE pathway was developed^20^. In this pathway, formaldehyde is first converted to formyl-CoA. One molecule formaldehyde and one molecule formyl-CoA are then condensed into the C2 compound glycolyl-CoA through either members of the 2-hydroxy-acyl-CoA synthase (HACL) or the oxalyl-CoA (OXC) family^20,21,32^. Glycolyl-CoA can be further valorized into a diverse set of molecules downstream.

**Fig. 5:**
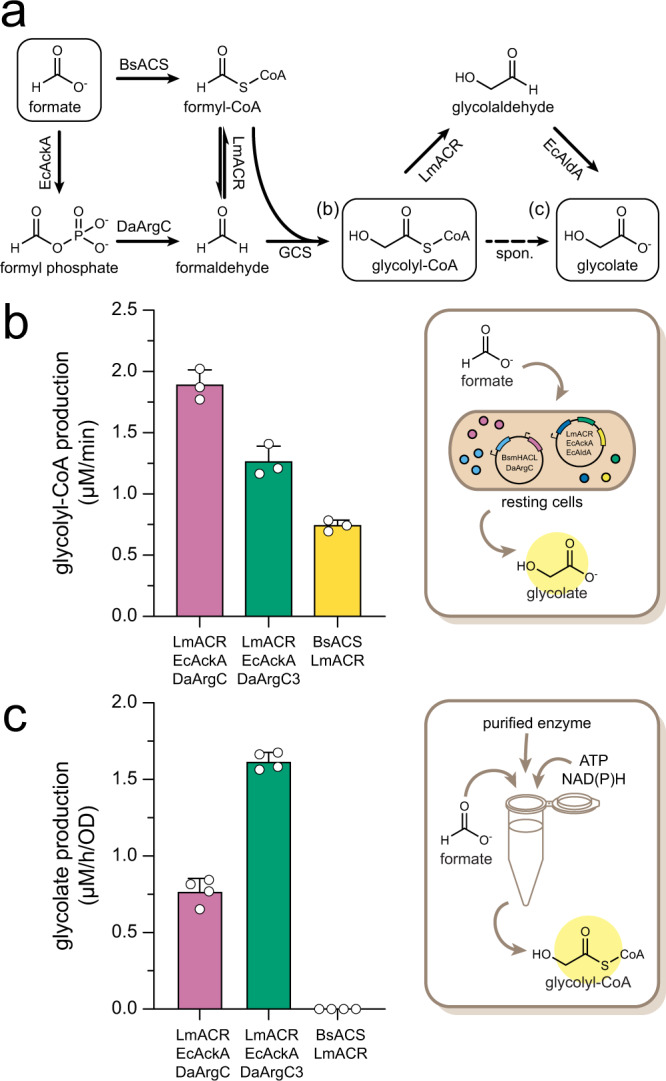
The P_i_ route can be integrated into the FORCE pathway to produce C2 compounds from formate. **a** Scheme of the formate activation/ reduction reactions required to produce formaldehyde and formyl-CoA to feed the downstream reactions of the FORCE pathway. **b** Glycolyl-CoA production by the P_i_ and CoA modules in vitro. Shown are single measurements, mean and standard error of technical replicates (*n* = 3). **c** Glycolate production by the P_i_ and CoA modules in resting cells after 3 h. Shown are single measurements, mean and standard error of biological replicates (*n* = 4). Negative controls as well as glycolate production at 3 and 24 h are shown in Supplementary Fig. 11. BsACS *Bacillus subtilis* acetyl-CoA synthetase, EcAckA *Escherichia coli* acetate kinase, DaArgC *Denitrovibrio acetiphilus N-*acetyl-γ-glutamyl phosphate dehydrogenase, LmACR *Listeria monocytogenes* acyl-CoA reductase, GCS glycolyl-CoA synthase, EcAldA *E. coli* glycolaldehyde dehydrogenase. Source data are provided as a Source Data file and in Edmond [10.17617/3.BKLI0C].

To test whether the P_i_ route was able to feed the FORCE pathway, we coupled EcAckA and WT DaArgC or DaArgC3 to acylating CoA-reductase LmACR (to convert formaldehyde into formyl-CoA) and engineered OXC-family member MeOXC4^32^ (for glycolyl-CoA formation from formaldehyde and formyl-CoA). As positive control, we used the published CoA route, in which BsACS + LmACR^19^ provide formaldehyde and formyl-CoA from formate (Fig. 5a). Notably, in this in vitro experiment, the WT DaArgC and the DaArgC3 route outperformed the CoA-based route by up to twofold, thus not only outperforming the current standard in the field not only thermodynamically, but also kinetically (Fig. 5b).

Both the WT DaArgC and the DaArgC3-dependent P_i_ routes produced glycolate in resting cell experiments(Fig. 5c) via downstream reduction of glycolyl-CoA^21^ (Fig. 5a). Notably, in this set-up, the CoA route yielded no product. Moreover and as expected, the DaArgC3-dependent P_i_ route outperformed the WT DaArgC-based route in vivo by approximately twofold, which supported the notion that our DaArgC3 engineering provided a specific benefit in vivo (but not in vitro).

Overall, this data demonstrated the successful operation of the FORCE pathway on formate as its sole carbon source, providing evidence for the thermodynamic and kinetic benefit of the P_i_ route for formate activation in vivo.

## Discussion

Here, we established a formyl phosphate route from formate to formaldehyde that involves two new-to-nature enzyme activities: FOK and FPR. To that end, we harnessed the latent formate kinase activity in EcAckA and detected FPR activity in four *E. coli* reductases; ArgC, GapA, ASD and ProA. Based on the favorable activities of ArgC with formyl phosphate, we focused on further improving this FPR activity. Phylogenetic screening identified a superior ArgC homolog from *D. acetiphilus* that we further enhanced by directed evolution. We employed random and iterative saturation mutagenesis to convert DaArgC into a bona fide FPR. During three rounds of mutagenesis, in vivo activity of variant DaArgC3 was improved fourfold over wild-type in vivo and a 300-fold selectivity switch for formyl phosphate over its native substrate was observed.

Although we observed increased activity of engineered DaArgC1-3 in vivo, this was not reflected in our in vitro measurements, where WT DaArgC still performed best. This can be simply explained by the fact that in vitro assays are performed in very controlled conditions that often ignore the complexity of in vivo metabolic networks. When feeding high concentrations (i.e., 50 mM formyl phosphate) to WT DaArgC, the enzyme outperforms the engineered variants DaArgC1-3 due to its superior *k*_cat_. However, in vivo, formyl phosphate concentrations are expected to be in the lower mM range, which benefits variants DaArgC1-3 that show an improved *K*_m, app_ compared to WT DaArgC. Even more importantly, engineered variants DaArgC1-3 show relaxed cofactor specificity, do not accept the natural substrate NAGP anymore, are not allosterically inhibited by NAG and also discriminate better against acetyl phosphate, which is an abundant metabolite in the cell. Thus, it is not so much the actual kinetics of the FRP reaction that become important in the cellular context, but the orthogonality of the enzyme in the metabolic network of *E. coli* that favor these variants in vivo.

The probably most limiting factor of the P_i_-based route in vivo is currently its side reactivity with acetate. While the FPR reaction in engineered variants DaArgC1-3 discriminates well against acetyl phosphate, EcAckA is a native enzyme that readily accepts acetate. A bona fide formate kinase would confer complete orthogonality, preventing metabolic crosstalk of the cascade. A specific formate kinase was described in the 1960s^23^ and might offer a solution, however, the corresponding gene has never been identified. Alternatively, AckA templates could be engineered towards a more efficient and specific formate kinase activity.

Through the P_i_ route, formaldehyde is readily produced from formate in vitro and in vivo at the cost of only one energy-rich phosphoanhydride bond and one reducing equivalent (i.e., NAD(P)H). Compared to previously published formate to formaldehyde conversion via formyl-CoA^15,17,19,21,22,33–35^, the formyl phosphate sequence requires one fewer energy-rich phosphoanhydride bond and operates entirely orthogonal to the acetyl-CoA pool. Compared to the natural formyl-T_4_F route^18^, the cascade does not need the complex T_4_F cofactor, requires fewer enzymes and does not depend on spontaneous hydrolysis, which makes the FOK-FPR cascade superior to both routes.

Recently, the landscape of synthetic formate conversion has been expanded by the FORCE^20^, HWLS^18^ and SMGF^19^ pathways that co-assimilate formate via T_4_F (HWLS) and CoA (FORCE, SMGF). Integration of FOK-FPR into these systems may relax protein burden and come at reduced thermodynamic cost (i.e., phosphoanhydride hydrolysis) and/or relieve kinetic constraints (i.e., spontaneous methylene-T_4_F hydrolysis). In a proof-of-principle, we showed that our cascade is able to feed the FORCE pathway^20^. Implementing the P_i_ route into the FORCE pathway outcompeted the CoA route both in vitro and in resting cells and demonstrated that the FORCE pathway can produce C2 compounds solely on formate as feedstock. With these encouraging results in mind, the P_i_ route may indeed prove beneficial to other synthetic formate conversion systems that require formaldehyde production.

Overall, our study extends the solution space of existing formate to formaldehyde conversion pathways and enriches the metabolic landscape of formate assimilation by another, thermodynamically efficient alternative, providing the basis for their subsequent in vivo implementation.

## Methods

### Chemicals

Chemicals were obtained from Merck KGaA (Darmstadt, Germany) and Carl Roth GmbH (Karlsruhe, Germany). Biochemicals and commercial enzymes were obtained from Thermo Fisher Scientific (Waltham, MA, USA) and New England Biolabs GmbH (Frankfurt am Main, Germany). Gel and plasmid purification kits were procured from Machery Nagel (Düren, Germany). A list of reagents and catalog numbers is supplied in Supplementary Data 1.

### Max-min driving force analysis

Max-min driving force (MDF) analysis^36^ was applied to evaluate the thermodynamics driving force of different formate reduction routes with python packages equilibrator_api (v 0.4.7) and equilibrator_pathway (v 0.4.7). The changes in Gibbs energy of the reactions were estimated using the component contribution method^37^, under the condition of pH 7.0, ionic strength of 0.25 M, and –log[Mg2+] (pMg) of 3. Cofactor and metabolite concentrations were constrained to the range 1 µM to 10 mM^36^. MDF values of different routes were computed for formate concentration between 1 and 50 mM. The scripts and details are available at Github (https://github.com/he-hai/PubSuppl) within the “2022_formate_reduction” directory.

### Flux balance analysis

Flux balance analysis (FBA) modeling was conducted with COBRApy (v 0.20.0)^38^ using the most updated *E. coli* genome-scale metabolic model iML1515^39^. The model was modified as follows: (i) transhydrogenase (THD2pp) translocates one proton instead of two^40^; (ii) homoserine dehydrogenase (HSDy) was set to irreversibly produce homoserine^14^; (iii) anaerobic relevant reactions, PFL, OBTFL, FDR2, and FDR3, were removed from the model; (iv) POR5, GLYCK, FDH4pp FDH5pp, GART, DRPA, and PAI2T were also knocked out to block unrealistic routes; (v) the formyl-CoA transferase reaction (FORCT) was blocked to compute the CoA route; (vi) the ATP maintenance reaction (ATPM) was also switched off.

To compare the potentials of the three formate reduction routes in biochemical production, the yields of the 12 building block precursors^41^ and biomass from formate were computed. The ribulose monophosphate (RuMP) cycle was employed as the formaldehyde assimilation pathway, as it has been successfully transplanted in *E. coli*^42,43^. The models of the three formate reduction routes and the RuMP pathway were integrated in the modified *E. coli* model. The maximum yields were estimated using formate as feedstock. The full code and models, including the changes described above, have been deposited to Github (https://github.com/he-hai/PubSuppl) within the “2022_formate_reduction” directory.

### Strains and plasmids

Strains and plasmids are listed in Supplementary Table 4 and Supplementary Data 2. Primers (Supplementary Data 3) were obtained from Eurofins MWG GmbH (Ebersberg, Germany). Gene fragments were ordered from TWIST Bioscience (San Francisco, CA, USA). Sequencing was performed by Microsynth Seqlab (Göttingen, Germany).

### Cloning

In vivo construct pCDFDuet-1_EcAckA_DaArgC was constructed using standard RELC. First, pCDFDuet-1 and pET28a_DaArgC were digested at 37 °C, 1 h with NdeI and XhoI in FastDigest buffer, with FastAP supplemented to pCDFDuet-1. Fragments were gel purified and ligated at 16 °C, overnight using T4 ligase in T4 ligase buffer. Ligation reactions were heat-inactivated at 65 °C and transformed into chemocompetent *E. coli* DH5α. Individual clones were evaluated for successful integration using Microsynth T7term primer. In the next step, RELC was performed with the resulting pCDFDuet-1_DaArgC and pCA24N_EcAckA using BamHI and HindIII as described above. Successful integration was evaluated by sequencing with DuetDOWN1 primer (AddGene, Watertown, MA, USA).

The primer and vector combinations for Golden Gate (GG) vector construction are given in Supplementary Data 4. GG parental vectors pCDFDuet-1_EcAckA_GFP and pET28a_GFP were constructed by Golden Gate cloning. Fragments were amplified using Q5 polymerase in Q5 buffer in a FlexCycler^2^ (Analytic Jena; Jena, Germany). Annealing temperatures were calculated by NEB Tm calculator v1.13.0. PCR product was purified and eluted in water. GG reactions were set up using T4 ligase and buffer as well as BsaI-HF V2. Cycles were 15 × 1.5 min, 37 °C and 3 min, 16 °C. After ligation, the reactions were kept at 16 °C. GG reaction was transformed into chemocompetent *E. coli* DH5α without purification. Successful integration was evaluated by green/white screening of the colonies and sequencing with Microsynth primer T7term. For GG constructs based on the parental constructs, gene fragments were either produced in the identical fashion or obtained with appropriate GG overhangs and codon-optimized for *E. coli* from TWIST Biosciences (San Francisco, CA, USA). In the GG reaction, after 15 cycles, T4 ligase was inactivated by heat-shock at 50 °C, 5 min, permitting removal of religated BsaI sites. Finally, BsaI was inactivated at 80 °C and the reactions were treated as described above.

For ISM, pCDFDuet-1_EcAckA_DaArgC was mutated using degenerated primers. During the course of the ISM, primers were adjusted to include the mutations found during screening. PCR reactions were run in a FlexCycler^2^ (Analytic Jena; Jena, Germany) using the saturation mutagenesis protocol for NEB Q5 polymerase and annealing temperatures calculated by the NEB Tm calculator v1.13.0. PCR products were gel purified and digested at 37 °C overnight using DpnI in FastDigest buffer. After PCR purification, the DNA was transformed into *E. coli* DH5α and evaluated for successful integration by sequencing with Microsynth T7term primer.

The template for random mutagenesis, DaArgC3_RDM_template, was constructed using a pET28a backbone by GG as depicted in Supplementary Data 4. From this template, DaArgC3 was randomized using primers RDM_DaArgC_fw and RDM_DaArgC_rv in a 50 µL reaction containing 1x Mg-free Taq buffer, 7 mM MgCl2, 50 µM MnCl2, 400 µM dATP and dGTP, 2 mM dTTP and dCTP, 400 µM of each primer, 5 ng/µL template and 1 µL Taq Polymerase. The PCR was cycled at 95 °C, 30 sec initial denaturation, 30 cycles of 95 °C, 30 s melting, 66 °C, 1 min annealing, and 68 °C, 2 min extension, with a final extension of 68 °C, 10 min. The product was PCR purified and directly used in a Golden Gate reaction with pCDFDuet-1_EcAckA_GFP. After transformation, 10 individual clones were sent for sequencing to determine the error rate. A library of a size of 2500 clones was generated by washing the transformation plates with LB and performing a pooled DNA extraction.

G182 was reconstructed in vectors pCDFDuet-1_EcAckA_DaArgC2 and pCDFDuet-1_EcAckA_DaArgC3 using primers DaArgC_S178V-G182_fw and DaArgC_S178V-G182_rv. Mutagenesis was performed as described for ISM. The resulting vectors pCDFDuet-1_EcAckA_DaArgC2* and pCDFDuet-1_EcAckA_DaArgC3* were used as template for onboarding of DaArgC2* and DaArgC3* into pET28a using the GG protocol given above.

### Protein purification

Proteins were produced in *E. coli* BL21 (DE3). Transformants were inoculated into 0.5 L TB supplemented with the appropriate antibiotic (50 µg/mL kanamycin for pET28a constructs, 34 µg/mL chloramphenicol for pCA24N constructs). Cells were grown at 37 °C to OD_600_ ~ 0.8, then cooled to 25 °C and induced by supplementation of 250 µL IPTG. After 16 h, cells were harvested by centrifugation (4500 × *g*, 10 min), resuspended in buffer A (500 mM KCl, 50 mM HEPES-KOH pH 7.8) and lysed by sonication with a SONOPULS HD 4000 with a KE 76 tip (BANDELIN electronic GmbH & Co. KG, Berlin, Germany) at 50% amplitude for 3 times 1 min of 1 s on/off pulses. Cell lysate was clarified by centrifugation at 75,000 × *g*, 4 °C for 45 min, then filtered through a 0.4 µm syringe tip filter (Sarstedt, Nümbrecht, Germany). Affinity purification was performed using 1 mL Ni-Sepharose Fast Flow column (HisTrap FF, GE Healthcare, Little Chalfont, UK) on an Äkta FPLC system from GE Healthcare (GE Healthcare, Freiburg, Germany). Filtered lysate was loaded onto columns equilibrated with buffer A, then washed with 85% buffer A, 15% buffer B (500 mM KCl, 50 mM HEPES-KOH pH 7.8, 500 mM imidazole). Protein was eluted with 100% buffer B in 1 mL fractions. Those containing protein were pooled and buffer exchange to storage buffer (150 mM KCl, 50 mM HEPES-KOH pH 7.8) was performed on two stacked 5 mL HiTrap desalting columns (GE Healthcare). Proteins were concentrated by ultrafiltration (Amicon Ultra) at 4000 × *g*. Concentration was determined on a NanoDrop 2000 Spectrophotometer (Thermo Scientific, Waltham, MA, USA) using the extinction coefficient at 280 nm, as calculated by protparam (https://web.expasy.org/protparam/). Enzyme purity was confirmed by SDS-PAGE. The purified proteins were flash-frozen in liquid nitrogen and stored at −80 °C.

### Phylogenetic analysis of the ArgC family

*E. coli*ArgC was analyzed using BLASTP v2.13.0^44^ and 500 homologs were exported. Using the hhfilter tool of HH-suite version 3^45^, all homologs with greater than 60% identity to the *E. coli* variant were excluded, resulting in 119 sequences. The tree was constructed in MEGA X v10.0.5^46^. The evolutionary history was inferred using the Neighbor-Joining method^47^. The bootstrap consensus tree inferred from 50 replicates was taken to represent the evolutionary history of the taxa analyzed^48^. Branches corresponding to partitions reproduced in less than 50% bootstrap replicates were collapsed. Evolutionary distances were computed using the Poisson correction method and are in the units of the number of amino acid substitutions per site. All ambiguous positions were removed for each sequence pair (pairwise deletion option). There were a total of 1203 positions in the final dataset.

### SDS-PAGE analysis of protein production

BL21 DE3 Δ*frmRAB* or BL21 DE3 Δ*frmRAB* Δ*pflAB* containing pCDFDuet-1_EcAckA_XxArgC were grown in 600 µL LB 50 µg/mL streptomycin in 2.0 mL 96-Deep Well Plates with V Bottom (Plate One) and grown overnight. The following day, the cultures were transferred 30 µL into 600 µL M9 20 mM glucose, 50 µg/mL streptomycin, grown for 2 h at 37 °C, cooled to 25 °C, induced with 250 µM IPTG and grown for 16 h at 25 °C. After expression, cells were harvested by centrifugation (11,000 × *g*) and resuspended in 50 µL H_2_O. Five microliters of the suspension were added to 5 µL of water and 10 µL of 5× SDS loading buffer and heated at 95 °C for 30 min. Ten microliters of the resulting sample were separated on a Mini-PROTEAN TGX gel (4–20%, BioRad; Hercules, CA, USA) in SDS-buffer and stained with Simply Blue SafeStain.

### Proteomic analysis of DaArgC variants

BL21 DE3 Δ*frmRAB* containing pCDFDuet-1_EcAckA_DaArgCx were grown in 20 mL LB 50 µg/mL streptomycin, induced with 250 µM IPTG and grown for 16 h at 25 °C. Cell pellets corresponding to OD = 1 were harvested by centrifugation (11,000 × *g*). Cells were lysed by incubation with 400 μL of a 2% sodium lauroyl sarcosinate (SLS) solution at 95 °C for 15 min and subsequent sonication (VialTweeter; Hielscher, Teltow, Germany). Cell lysates were then reduced by the addition of 5 mM tris(2-carboxyethyl)phosphine and incubation at 95 °C for 15 min, followed by alkylation (10 mM iodoacetamide for 30 min at 25 °C). The cell lysates were cleared by centrifugation, and the total protein was estimated for each sample with a Pierce bicinchoninic acid (BCA) protein assay kit (Thermo Fisher Scientific, Waltham, MA, USA). The cell lysate containing 50 μg of total protein was then diluted with 100 mM ammonium bicarbonate to a final detergent concentration of 0.5% and digested with 1 μg of trypsin (Promega, Madison, WI, USA) overnight at 30 °C. Next, SLS was removed by precipitation with 1.5% trifluoroacetic acid (TFA) and centrifugation (11,000 × *g*). Peptides were purified using C18 microspin columns according to the manufacturer’s instructions (Harvard Apparatus, Holliston, MA, USA).

Purified peptides were dried, resuspended in 50 μL of 0.1% TFA, and analyzed by liquid chromatography–mass spectrometry (MS) carried out on a Exploris 480 instrument connected to an Ultimate 3000 rapid-separation liquid chromatography (RSLC) nano instrument and a nanospray flex ion source (all Thermo Fisher Scientific, Waltham, MA, USA). Peptide separation was performed on a reverse-phase high-performance liquid chromatography (HPLC) column (75 μm by 42 cm) packed in-house with C18 resin (2.4 μm; Dr. Maisch HPLC GmbH, Ammerbuch, Germany). The peptides were first loaded onto a C18 precolumn (preconcentration set-up) and then eluted in the backflush mode with a gradient from 98% solvent A (0.15% formic acid) and 2% solvent B (99.85% acetonitrile and 0.15% formic acid) to 25% solvent B over 65 min, continued from 25 to 35% of solvent B for another 24 min. The flow rate was set to 300 nL/min. The data were acquired in a data-independent mode (DIA) for the initial label-free quantification, and study was set to obtain one high-resolution MS scan at a resolution of 120,000 (*m/z* 200) with the scanning range from 320 to 1400 *m/z* followed by DIA scans with 27 fixed DIA windows with the width of 24 *m/z* (1 *m/z* overlap from neighboring windows), ranging from 320 to 970 *m/z* at a resolution of 15,000. The automatic gain control was set to 300% for MS survey scans and 3000% for DIA scans.

Spectra were identified with the tool DIA-NN^49^ for extracting peptide signals from raw files using a library-free search with a custom *E. coli* database. Sequences of over-expressed proteins were provided. To the UniProt *E. coli*K12 reference proteome we added DaArgC and EcAckA as custom proteins (Supplementary Fig. 20). EcAckA should be present in all samples. DaArgC had short sequences removed (always on the tryptic lysine residue), that covered regions with point mutations in different variants (Supplementary Fig. 20). Over 90% of peptides measured by the mass spectrometer accounts for the shared sequence, and we used that peptides for quantification of different isoforms in different samples. Based on the experimental design, conditions should contain only a single isoform. DIA-NN was used with settings included in report.log.txt. In brief, output was filtered at 0.01 FDR. Deep learning was used to generate a new in silico spectral library. Library-free search was enabled. Min fragment *m/z* set to 200. Max fragment *m/z* set to 1800. *N-*terminal methionine excision enabled. In silico digest involves cuts at K,R Maximum number of missed cleavages set to 1. Min peptide length set to 7. Max peptide length set to 30. Min precursor *m/z* set to 320. Max precursor *m/z* set to 950. Min precursor charge set to 1. Max precursor charge set to 4. Cysteine carbamidomethylation enabled as a fixed modification. Maximum number of variable modifications set to 2. Modification UniMod:35 with mass delta 15.9949 at M will be considered as variable. Protein x sample matrices will be filtered using run-specific protein q-value. DIA-NN will optimize the mass accuracy automatically using the first run in the experiment.

Data analysis and statistic were carried out by the Autonomics package developed in-house (10.18129/B9.bioc.autonomics) based on the “report.tsv” of the DIA-NN spectral identification output. Proteins with a protein *q* value of <0.01 were included for further analysis.

### Crystallization of DaArgC and DaArgC3

DaArgC and DaArgC3 were purified as described above. Immediately after affinity purification, the eluate was loaded onto a HiLoad 16/600 Superdex 200 pg column (GE Healthcare; Chicago, IL, USA) equilibrated in SEC buffer (75 mM KCl, 25 mM HEPES-KOH pH 7.8). Fractions corresponding to dimeric enzyme were collected, pooled and concentrated to 10 mg/mL on 10,000 MWCO Amicon Ultra filters (Merck KGaA; Darmstadt, Germany) by centrifugation (4000 x g). Enzyme purity was evaluated via SDS page. Crystal plates were set up using the sitting drop vapor diffusion method, diluting equal volume of enzyme in reservoir solution. The reservoir solution for DaArgC contained 0.2 M potassium bromide, 0.2 M potassium thiocyanate, 0.1 M sodium cacodylate pH 6.5, 3 % w/v γ-PGA (Na^+^ form, LM) and 2 % v/v PEG 500 MME and the reservoir for DaArgC3 contained 0.2 M sodium chloride, 0.1 M HEPES pH 7.5 and 25%(w/v) PEG 4000.

X-ray diffraction data for DaArgC and DaArgC3 were collected at the Beamline ID30B using MxCube3 v2.3.0.1 (European Synchrotron Radiation Facility, Grenoble, France). All images were processed using XDS Built=20200417^50^. The datasets were scaled using SCALA v3.3.22 of the CCP4 program suite^51^. The Phenix v1.18.2-3874 software package^52^ was used to perform molecular replacement (PhaserMR v2.8.3) for phasing of both datasets by using the ArgC from *Shigella flexneri* (PDB 3dr3) as search model^53^. Initial models were built with phenix.autobuild and refined with the phenix.refine and Coot^54^. Data collection and refinement statistics are provided in Supplementary Table 5.

### Formyl phosphate synthesis

To an aqueous solution of dipotassium phosphate (1 mL, 1 M, 1 equiv.), acetic formic anhydride (0.106 g, 1.2 equiv.) was added (For synthesis of formic acetic anhydride, see Supplementary Method 1 and Supplementary Fig. 12). The solution was mixed shortly and monitored by ^31^P{^1^H}-NMR. The first measurement after 1 minute shows that 59% of dipotassium phosphate was converted to formyl phosphate. (Supplementary Method 2 and Supplementary Figs. 13–15).

The decomposition of formyl phosphate was carried out under similar conditions applied in the enzymatic process. An aliquot of a solution of dipotassium phosphate prepared as described above (0.1 mL) was added to a solution of MOPS buffer 0.5 M and magnesium chloride 0.01 M (0.9 mL) mixed shortly and directly monitored via ^31^P{^1^H}-NMR. The concentration of formyl phosphate linearly decreased at a rate of −0.34 %min^−1^ (Supplementary Method 3 and Supplementary Fig. 16).

### NMR analysis

^1^H-NMR measurements for the formic acetic anhydride synthesis were conducted at room temperature on a Bruker AS 400 (Bruker Corporation, Billerica, MA, USA) spectrometer. The chemical shift δ is given in ppm and was referenced to the solvent residual signal. ^1^H- and ^31^P-measurements for the formyl phosphate formation and decomposition were conducted at 30 °C on a Bruker AV 300 spectrometer.

### Spectrometric analysis

Activity of AckAs on formate and DaArgC on *N-*acetyl-γ-glutamyl phosphate was determined in 100 µL of reaction volume in a High Precision Cell 10 mm Light Path quartz cuvette (Hellma Analytics; Biel-Benken, Switzerland) using a CARY 60 UV–Vis Spectrophotometer (Agilent; Santa Clara, CA, USA) Kinetic program. Unless otherwise mentioned, components were added to the cuvette in the order that they are listed, and the last indicated component was used to start the reaction. NAD(P)H consumption or production was assessed by tracking the absorbance at 340 nm in 0.5 s intervals over time. Data was fit using the linear regression tool of the Cary WinUV Kinetics application v5.0.0. Mean and standard deviation as well as Michaelis Menten fit were calculated in GraphPad Prism v9.0.2 using standard settings.

AckA kinetics on formate were determined at 37 °C. Reactions contained 100 mM HEPES-KOH pH 7.5, 10 mM MgCl_2_, 1 mM ATP, 250 µM NADH, 5 mM PEP, 1:40 PK/LDH mix and 0.1 µM AckA. Ammonium formate concentrations were 3, 10, 20, 50, 150 and 300 mM. Initial velocities were determined and a Michaelis Menten kinetic was constructed.

Activity of DaArgC on *N-*acetyl-γ -glutamyl phosphate was measured at 30 °C in reactions containing 100 mM MOPS pH 7.0, 10 mM MgCl_2_, 5 mM ATP, 250 µM NADPH,1 µM ArgB, 0.1 µM DaArgC (20 µM DaArgC1-3) and 50 mM *N-*acetyl-γ-glutamic acid neutralized with KOH.

Activity of DaArgC on acetyl phosphate was carried out at 30 °C in an Infinite M PLEX plate reader (Tecan) on half-well plates (Greiner) with a liquid column height of 4 mm. Reactions contained 100 mM MOPS pH 7.0, 10 mM MgCl_2_, 250 µM NADPH, 1 µM DaArgC and 50 mM acetyl phosphate.

### Formaldehyde detection via Nash assay

Formaldehyde was detected by reaction with the Nash reagent: 2 M ammonium acetate, 20 mM acetyl acetone and 50 mM acetic acid. Samples were diluted 1:1 into the reagent. Where required, a formaldehyde calibration curve in the sample matrix was added for quantification. After incubation for 1 h at 37 °C, the brightly yellow diacetyldihydrolutidine was formed, which was detected by fluorescence measurements at 412 nm excitation/ 505 nm emission in both 96-well and 384-well formats in an Infinite M PLEX plate reader (Tecan; Männedorf, Switzerland). Side reactivity with formyl phosphate, formyl-CoA or acetaldehyde was not observed (Supplementary Fig. 21). Data was collected using Tecan iControl v3.8.2.0 and Microsoft Excel (v2208). Using GraphPad Prism v9.0.2, data was quantified against the formaldehyde calibration curve and, where relevant, a linear fit of the individual measurements was applied using the standard settings of the GraphPad Prism tool. Mean and standard deviation were calculated by the program.

In vitro reactions were carried out at 30 °C and started by addition of formate. Samples were taken in regular intervals as indicated below. Samples were quenched 1:1 in 10% formic acid, then one volume equivalent of Nash reagent was added for detection.

Formyl phosphate reductases were compared in reactions containing 100 mM MOPS-KOH pH 7.0, 10 mM MgCl_2_, 5 mM ATP, 1 mM CoA, 250 µM NAD(P)H, 50 mM ammonium formate, 2 µM EcAckA and 2 µM of ArgC, ASD, GapA or ProA. Time points were taken at 1, 5, 10 and 15 min.

ArgC homologs were compared in reactions containing 100 mM MOPS-KOH pH 7.0, 10 mM MgCl_2_, 5 mM ATP, 250 µM NADPH, 50 mM ammonium formate, 1 µM EcAckA and 5 µM ArgC. Time points were taken at 1.5, 5, 10 and 20 min.

DaArgC. was characterized in reactions contained 100 mM MOPS-KOH pH 7.0, 10 mM MgCl_2_, 5 mM ATP, 250 µM NADPH, 200 mM ammonium formate (and/or 200 mM ammonium acetate), 10 µM EcAckA and 1 µM DaArgC (if doubled; 20 or 2 µM enzyme). Time points were taken at 0.5, 1, 2 and 3 min.

In vitro variant activity was determined in reactions of 100 mM MOPS-KOH pH 7.0, 10 mM MgCl_2_, 5 mM ATP, 250 µM NADPH, 50 mM ammonium formate, 2 µM EcAckA and 2 µM DaArgC. Time points were taken at 1, 5, 10 and 15 min.

For the kinetic analysis of DaArgC variants, initial slopes were determined and Michaelis Menten plots were constructed. Assessment of formyl phosphate kinetics was carried out as follows: formyl phosphate was produced as described above. The synthesis was incubated for 1 min before being added 1:10 into the enzymatic reaction containing 500 µM MOPS pH 7.0, 10 mM MgCl_2_, 1 mM NADPH and 2 µM DaArgC variant. Time points were taken at 1, 2, 3 and 5 min. Assessment of NADPH kinetics was carried out as follows: reactions contained 100 mM MOPS-KOH pH 7.0, 10 mM MgCl_2_, 5 mM ATP, 500 mM ammonium formate, 10 µM EcAckA and 1 µM of DaArgC variants. NADPH was added in the range of 2.5 µM up to 500 µM. Time points were taken at 1, 2 and 3 min for DaArgC, DaArgC2* and DaArgC3* and 1, 3 and 5 min for DaArgC1-3. Assessment of NADH kinetics was carried out as follows: reactions contained 100 mM MOPS-KOH pH 7.0, 10 mM MgCl_2_, 5 mM ATP, 250 mM ammonium formate, 10 µM EcAckA and 1 µM of DaArgC variants NADH was added in the range of 10 µM up to 2000 µM. Time points were taken at 1, 4 and 8 min. Initial slopes were calculated and Michaelis Menten plots were constructed.

Inhibition of FPR activity by *N-*acetyl-γ-glutamic acid was determined in reactions containing 100 mM MOPS-KOH pH 7.0, 10 mM MgCl_2_, 5 mM ATP, 250 µM NADPH or 250 µM NADH, 200 mM ammonium formate, 10 µM EcAckA and 1 µM DaArgC and 0–1 M *N-*acetyl-γ-glutamic acid neutralized with KOH. Time points were taken at 1.5, 3 and 5 min.

For the determination of lysate activity of EcAckA and DaArgC, pCDFDuet-1_EcAckA_DaArgC was transformed into BL21 DE3 Δ*frmRAB*. The next day, 30 mL overexpression culture was grown to OD in LB at 37 °C, cooled to 25 °C, induced by addition of 250 µM IPTG and incubated overnight at 25 °C. The next day, the culture was spun down and the resulting pellet resuspended in 500 µL CellLytic B Cell Lysis Reagent. After incubation at room temperature for 10 min, the cell debris was spun down and the lysate stored on ice. Formaldehyde production was assayed in reactions containing 100 mM MOPS-KOH pH 7.0, 10 mM MgCl_2_, 5 mM ATP, 250 µM NADPH, 50 mM formate and 1:10 lysate. Two controls were additionally supplemented with 5 µM purified EcAckA or DaArgC. Time points were taken at 1, 5, 10 and 30 min.

In vivo reactions were carried out by transformation of pCDFDuet-1 and pCDFDuet-1_EcAckA_DaArgCx into BL21 DE3 Δ*frmRAB* or BL21 DE3 Δ*frmRAB* Δ*pflAB*. LB overnight cultures were set up in triplicates. The next day, 600 µL M9 + 20 mM glucose was inoculated with 30 µL overnight culture and grown on 2.0 mL 96-Deep Well Plates with V Bottom (Plate One; Munich, Germany) for 2 h at 37 °C. Cells were induced by addition of 250 µM IPTG, and incubated 16 h at 25 °C. The next day, formate was added to the reaction, incubated at 30 °C and time points were taken as indicated, stored at −20 °C and then quantified.

For the in vivo activity of pCDFDuet-1_EcAckA_DaArgC, 50 mM ammonium formate was added to the cells. Time points were taken at 0, 1, 2 and 4 h. The identical method was applied to evaluate the in vivo activity of the ArgC homologs.

For the ISM of DaArgC, single colonies of transformed ISM libraries were directly inoculated into 600 µL M9 + 20 mM glucose and grown for 8 h at 37 °C before proceeding as described above. 50, 25 or 5 mM of ammonium formate were added to the supernatant. Samples were taken at 2 or 4 h.

For the RDM of DaArgC, libraries and controls were transformed into BL21 DE3 Δ*frmRAB*. Media were dispensed using an automatic set-up built and tested in collaboration with Festo SE & Co. KG (Esslingen am Neckar, Germany) (Supplementary Fig. 22). Using a PIXL Colony Picking Robot (Singer Instruments; Roadwater, UK), cell were picked into 100 µL LB 50 µg/mL Kanamycin, 50 µg/mL streptomycin in a 384-well format. Cells were grown overnight at 1000 rpm in an Infors Multitron incubator (Bottmingen, Switzerland) set to 80% humidity. From the master plate, 30 µL culture were transferred into 600 µL M9 50 µg/mL Kanamycin, 50 µg/mL streptomycin + 20 mM glucose and at 37 °C for 2 h on 2.0 mL 96-Deep Well Plates with V Bottom (Plate One; Munich, Germany). Cultures were induced with 250 µM IPTG and incubated 16 h at 25 °C. 5 mM ammonium formate was added and samples were taken at 4 h.

To determine the in vivo activity of the DaArgC variants, 500, 50, 5 or 0 mM ammonium formate were added to the cells. Time points were taken at 0, 1, 2 and 4 h. Slopes were calculated using linear regression.

### LC–MS detection of glycolyl-CoA production

Glycolyl-CoA production was detected by LC-MS. Reactions contained 100 mM MOPS-KOH pH 7.0, 10 mM MgCl2, 150 µM TPP, 0.5 mM ADP, 5 mM ATP, 0.5 mM CoA, 250 µM NADPH, NAD+ and/or NADH and/or 0.5 mM K_x_H_y_PO_4_ pH 6.9, 10 µM MeOXC4 and 5 µM of LmACR, EcAckA, DaArgC, and/ or BsACS as well as 200 mM NH_4_ formate. Incubation occurred at 30 °C and time points were taken at 5, 20, 40 and 60 min by quenching of the reaction mixture 1:2 with 7.5 % formic acid.

Quantitative determination of glycolyl-CoA was performed using a LC-MS/MS. The chromatographic separation was performed on an Agilent Infinity II 1290 HPLC system using a Kinetex EVO C18 column (150 × 1.7 mm, 3 μm particle size, 100 Å pore size, Phenomenex) connected to a guard column of similar specificity (20 × 2.1 mm, 5 μm particle size, Phenomoenex) a constant flow rate of 0.25 mL/min with mobile phase A being 0.1 % formic acid in water and phase B being 0.1 % formic acid in methanol (Honeywell, Morristown, NJ, USA) at 40 °C. The injection volume was 2 µL. The mobile phase profile consisted of the following steps and linear gradients: 0–1 min constant at 5 % B; 1–4.5 min from 5 to 80 % B; 4.5–5.5 min constant at 80 % B; 5.5–5.6 min from 80 to 5 % B; 5.6 to 8 min constant at 5 % B. An Agilent 6470 mass spectrometer was used in positive mode with an electrospray ionization source and the following conditions: ESI spray voltage 5500 V, nozzle voltage 500 V, sheath gas 400 °C at 11 L/min, nebulizer pressure 20 psig and drying gas 100 °C at 5 L/min. Compounds were identified based on their mass transition and retention time compared to standards. Chromatograms were integrated using Agilent MassHunter software. Absolute concentrations were calculated based on an external calibration curve prepared in sample matrix. Mass transitions, collision energies, Cell accelerator voltages and Dwell times have been optimized using chemically pure standards. Parameter settings are given in Supplementary Table 6.

The resulting data was evaluated by integration in Agilent MassHunter Quantitative Analysis (QQQ) v10.0 and quantified using a glycolyl-CoA standard run with the same settings. Productivity was determined using a linear fit of the individual measurements in GraphPad Prism v9.0.2. Mean and standard deviation were calculated by the program.

### Resting cell bioconversion

In vivo product synthesis was conducted using M9 minimal media (6.78 g/L Na_2_HPO_4_, 3 g/L KH_2_PO_4_, 1 g/L NH_4_Cl, 0.5 g/L NaCl, 2 mM MgSO_4_, 100 μM CaCl_2_, and 15 µM thiamine-HCl) unless otherwise stated. Cells were initially grown in 96-deep well plates (USA Scientific, Ocala, FL) containing 0.5 mL of the above media further supplemented with 20 g/L glycerol, 10 g/L tryptone, and 5 g/L yeast extract. A single colony of the desired strain (Supplementary Table 7) was cultivated overnight (14–16 h) in LB medium with appropriate antibiotics and used as the inoculum (1%). Antibiotics (100 μg/mL carbenicillin, 100 μg/mL spectinomycin) were included when appropriate. Cultures were then incubated at 30 °C and 1000 rpm in a Digital Microplate Shaker (Fisher Scientific) until an OD_600_ of ~0.4 was reached, at which point appropriate amounts of inducer(s) (IPTG or cumate) were added. Plates were incubated for a total of 24 h post inoculation.

Cells from the above pre-cultures were then centrifuged (1500 × *g*, 22 °C), washed with the above minimal media without any carbon source, and resuspended with 1 mL of above minimal media containing indicated amounts of carbon source. 50 mM formate was added and incubated at 30 °C and 1000 rpm in Digital Microplate Shaker (Fisher Scientific). After incubation at 30 °C for 3 or 24 h, the cells were pelleted by centrifugation and the supernatant was analyzed by HPLC. Quantification of product and substrate concentrations (formic acid, formaldehyde and glycolic acid) were determined via HPLC using a Shimadzu Prominence SIL 20 system (Shimadzu Scientific Instruments, Inc., Columbia, MD) equipped with a refractive index detector and an Shim-pack Fast-OA column (Shimadzu) with operating conditions to optimize peak separation (0.4 mL/min flow rate, 5 mM *p*-toluenesulfonic acid mobile phase, column temperature 45 °C).

### Reporting summary

Further information on research design is available in the Nature Portfolio Reporting Summary linked to this article.

## Supplementary information

Supplementary Information

Description of Additional Supplementary Files

Supplementary Data 1

Supplementary Data 2

Supplementary Data 3

Supplementary Data 4

Reporting Summary

## Data Availability

Large photometric datasets (kinetic analysis of AckA homologs) and LC-MS measurements are deposited in Edmond [10.17617/3.BKLI0C]. X-ray structures can be accessed on PDB under accession codes 8AFU (DaArgC) and 8AFV (DaArgC3). Proteomic data is available on MassIVE under ProteomeXchange accession code PXD041037 Source data is provided with this paper. Source data are provided with this paper.

## Source data

Source Data

## Code availability

The scripts and details of MDF and FBA calculations are available at Github [https://github.com/he-hai/PubSuppl] within the “2022_formate_reduction” depository and can be found under 10.5281/zenodo.7752828^55^. Data analysis tools for proteomic analysis can be found under 10.18129/B9.bioc.autonomics^56^.
