# Supplementary material for: Engineering a new-to-nature cascade for phosphate-dependent formate to formaldehyde conversion in vitro and in vivo
Source: Nat Commun. 2023 May 9;14:2682. doi: 10.1038/s41467-023-38072-w (PMC10170137; doi:10.1038/s41467-023-38072-w)
Supplement: Supplementary file 1 — Supplementary Information [file 41467_2023_38072_MOESM1_ESM.pdf]

Engineering a new-to-nature cascade for phosphate-dependent formate to formaldehyde conversion *in vitro* and *in vivo*

Nattermann *et al.*

Supplementary Method 1. Synthesis of formic acetic anhydride

The synthetic procedure described hereafter has been carried out under an argon atmosphere using Schlenk-technique according to a literature procedure.[1] Briefly, sodium formate (1.1 eq., 220 mmol, 14.96 g) was dispersed in dry diethyl ether (50 mL) and cooled to 0 °C. To this slurry, acetyl chloride (1 eq., 200 mmol, 15.698 g) was added dropwise over 25 minutes. The mixture was vigorously stirred at 0 °C for 32 h till acetyl chloride was completely consumed as confirmed by monitoring the reaction via 1H-NMR. The mixture was filtered and the solid residue washed once with diethyl ether (10 mL). Filtrate and washing solution were combined and the volatiles removed at 0 °C under reduced pressure of 50 mbar. Acetic formic anhydride was obtained as colorless liquid (12.96 g) with a purity of 90.6 % as determined by NMR (impurities: acetic anhydride (3.9 %), acetic acid (1.2 %) and diethyl ether (4.7 %)) (Supplementary Figure 11).

Supplementary Method 2. Formyl phosphate synthesis

To an aqueous solution of dipotassium phosphate (1 mL, 1 M, 1 equiv.), acetic formic anhydride (0.106 g, 1.2 equiv.) was added. The solution was mixed shortly and monitored by $^{31}P\{^{1}H\}$-NMR. The first measurement after 1 minute shows that 59% of dipotassium phosphate was converted to formyl phosphate. The concentration of formyl phosphate linearly decreases at a rate of -1% min^{-1} (Supplementary Fig. 12). At the end of the reaction, the pH of the solution was around 4.

The integral ratio of the $^{31}P\{^{1}H\}$-NMR signals was used for quantification. At 0.1 ppm the singlet from dipotassium phosphate is detected, while formyl phosphate shows a singlet at -2.1 ppm (Supplementary Fig. 13). This assignment was confirmed by ^{31}P-NMR where formyl phosphate shows a doublet at -2.1 ppm (J_{PH}=5.8 Hz) and in ^{1}H-NMR a doublet at 8.3 ppm (J_{PH}=5.8 Hz) for the formyl group (Supplementary Fig. 3). ^{1}H-^{31}P-HMBC-NMR confirmed this assignment (Supplementary Fig. 14). Both doublets become singlets in the respective $^{1}H\{^{31}P\}$ and $^{31}P\{^{1}H\}$-NMR decoupled spectra (Supplementary Fig. 13).

Supplementary Method 3. Hydrolysis of formyl phosphate under reaction conditions

The decomposition of formyl phosphate under similar conditions applied in the enzymatic process was investigated. An aliquot of a solution of formyl phosphate prepared as described above (0.1 mL) was added to a solution of MOPS buffer 0.5 M and magnesium chloride 0.01 M (0.9 mL) mixed shortly and directly monitored via $^{31}P\{^{1}H\}$-NMR. The concentration of formyl phosphate linearly decreased at a rate of -0.34 %min^{-1}. This experiment confirms that the hydrolytic decomposition is negligible as compared to enzymatic reaction.

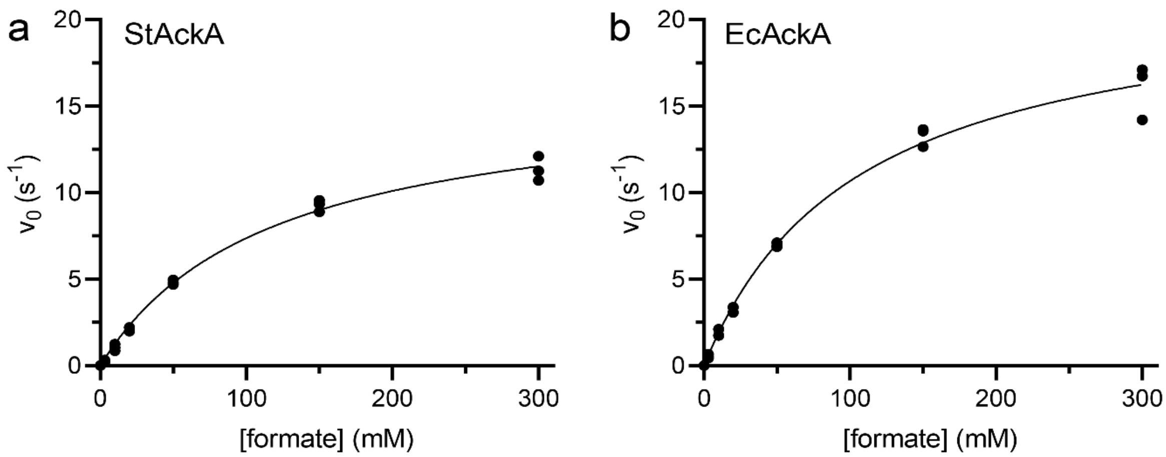

Supplementary Figure 1. Kinetics for formyl-phosphate synthesis from formate by acetate kinases. a Kinetic determination of *S. typhimurium* acetate kinase (StAckA). **b** Kinetic determination of *E. coli* acetate kinase (EcAckA). Initial velocity was determined and a Michaelis Menten kinetic was constructed. Individual technical replicates are shown (n=3). Source data are provided as a Source Data file and deposited in Edmond [https://doi.org/10.17617/3.BKLI0C].

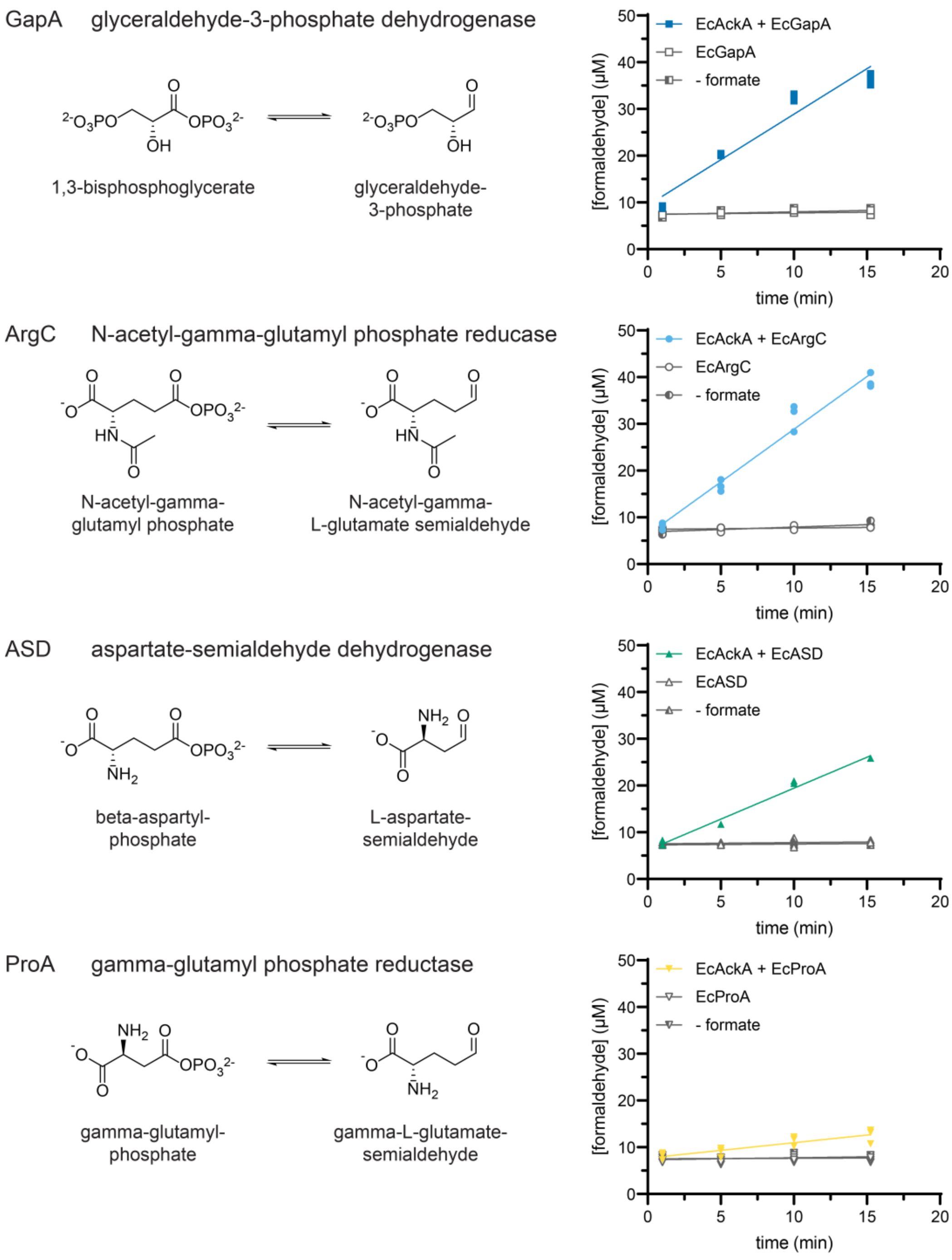

Supplementary Figure 2. Native function and promiscuous activities of formyl phosphate reductase candidates. Native reactions are given. Graphs depict formyl phosphate reductase activity and relevant controls. Formyl phosphate was produced *in situ* by *E. coli* acetate kinase (EcAckA). Shown is the linear fit of individual technical replicates (n =3). Source data are provided as a Source Data file.

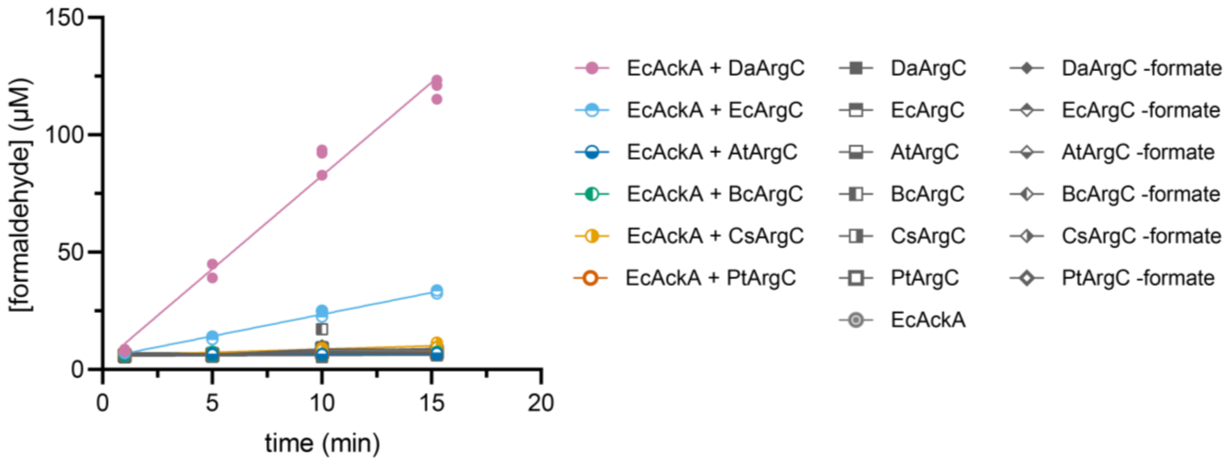

Supplementary Figure 3. Formaldehyde production by ArgC homologs. Formaldehyde production by EcAckA+XxArgC *in vitro* including relevant controls. Formaldehyde was detected by Nash assay. Shown is the linear fit of independent technical triplicates (n=3). EcAckA: *Escherichia coli* acetate kinase, AtArgC: *Arabidopsis thaliana N*-acetyl-γ-glutamyl phosphate dehydrogenase, BcArgC: *Bacillus clausii N*-acetyl-γ-glutamyl phosphate dehydrogenase, CsArgC: *Caldicellulosiruptor saccharolyticus N*-acetyl-γ-glutamyl phosphate dehydrogenase, DaArgC: *Denitrovibrio acetiphilus N*-acetyl-γ-glutamyl phosphate dehydrogenase, EcArgC: *Escherichia coli N*-acetyl-γ-glutamyl phosphate dehydrogenase, PtArgC: *Pseudidiomarina taiwanensis N*-acetyl-γ-glutamyl phosphate dehydrogenase. Source data are provided as a Source Data file.

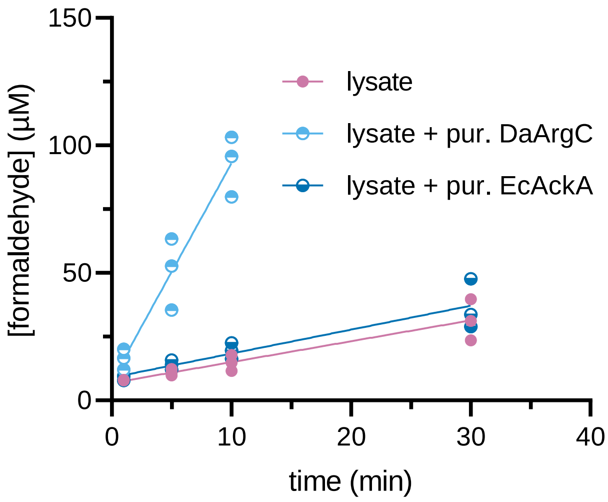

Supplementary Figure 4. Lysate activity of EcAckA+DaArgC. Activity of EcAckA+DaArgC in bacterial lysate. Shown are productivities with and without addition of 5 µM purified enzyme. Shown are a linear fit and individual measurements of independent biological replicates (n=3). EcAckA: *Escherichia coli* acetate kinase, DaArgC: *Denitrovibrio acetiphilus N*-acetyl-γ-glutamyl phosphate dehydrogenase. Source data are provided as a Source Data file.

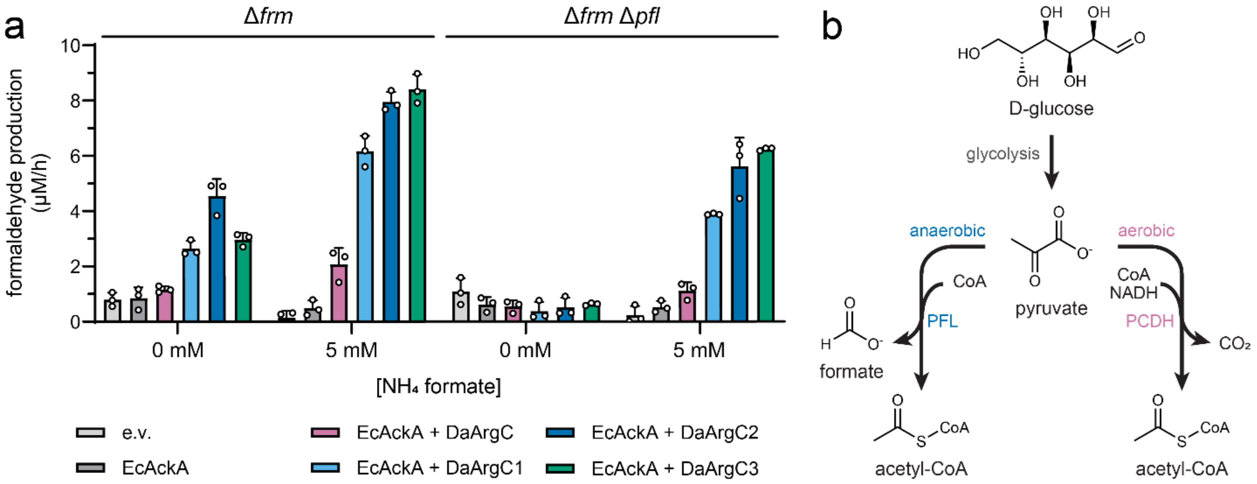

Supplementary Figure 5. Background formate production by PFL in Bl21 DE3. a Background formaldehyde production in BL21 DE3 Δ*frmRAB* and BL21 DE3 Δ*frmRAB* Δ*pflAB* on 0 mM and 5 mM formate. Shown are mean with standard deviation and individual measurements. Data points represent independent biological replicates (n=3). **b** Metabolic network of *E. coli* from glucose to acetyl-CoA. In anaerobic conditions, PFL produces formate and acetyl-CoA from pyruvate. EcAckA: *Escherichia coli* acetate kinase, DaArgC: *Denitrovibrio acetiphilus N*-acetyl-γ-glutamyl phosphate dehydrogenase. PFL: pyruvate formate lyase. PCDH: pyruvate dehydrogenase. Source data are provided as a Source Data file.

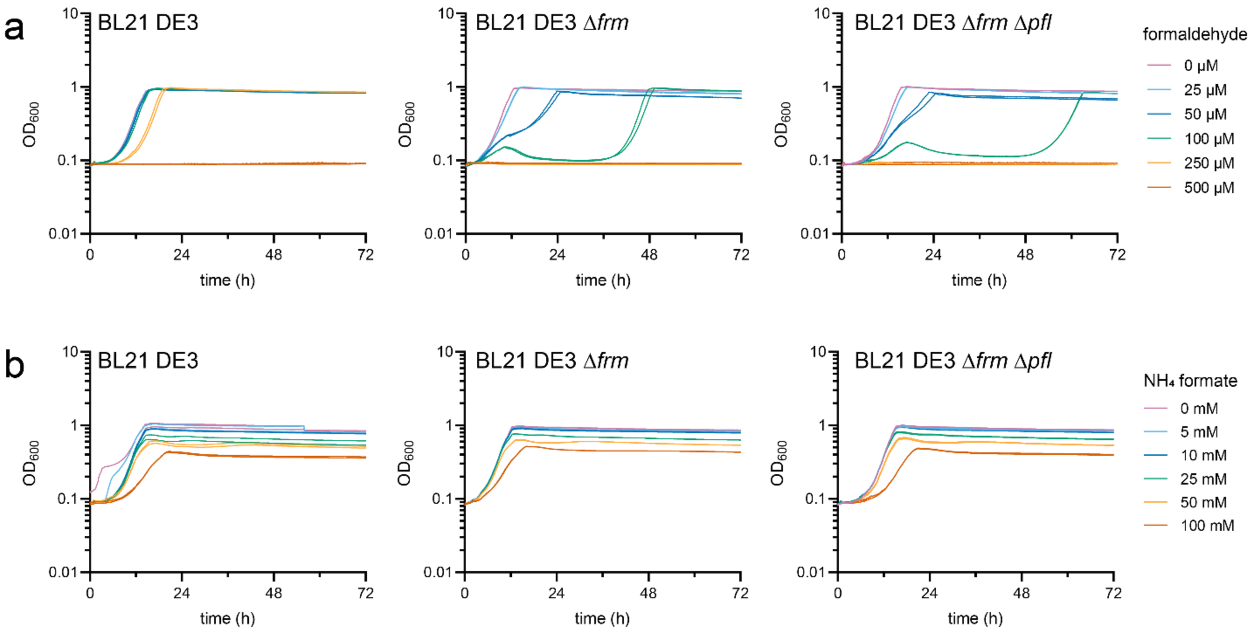

Supplementary Figure 6. Cell survival in the presence formate and formaldehyde. Toxicity of formaldehyde (**a**) and ammonium formate (**b**) on the growth of *E.coli* BL21 DE3 deficient in formaldehyde detoxification system *frmRAB* and/or pyruvate formate lyase *pflAB*. Shown are individual independent biological replicates (n=2). Source data are provided as a Source Data file.

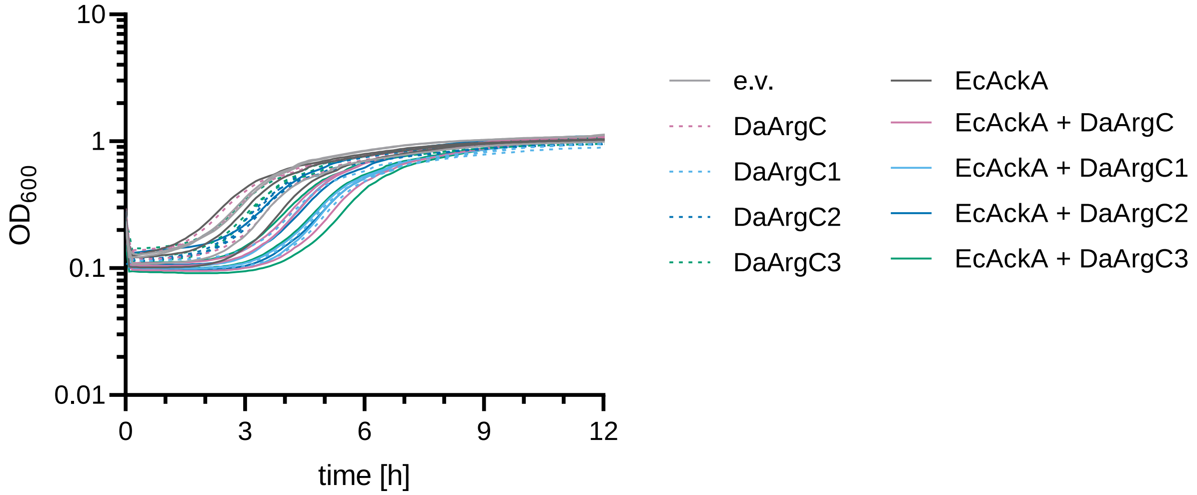

Supplementary Figure 7. Rescue of cells after assay. Rescue in LB of BL21DE3 Δ*frmRAB* cells carrying EcAckA and/or DaArgC variants after assay conditions (overexpression followed by 4 h incubation with 50 mM formate). Shown are mean and standard deviation of independent biological replicates (n=3). EcAckA: *Escherichia coli* acetate kinase, DaArgC: *Denitrovibrio acetiphilus N*-acetyl-γ-glutamyl phosphate dehydrogenase. Source data are provided as a Source Data file.

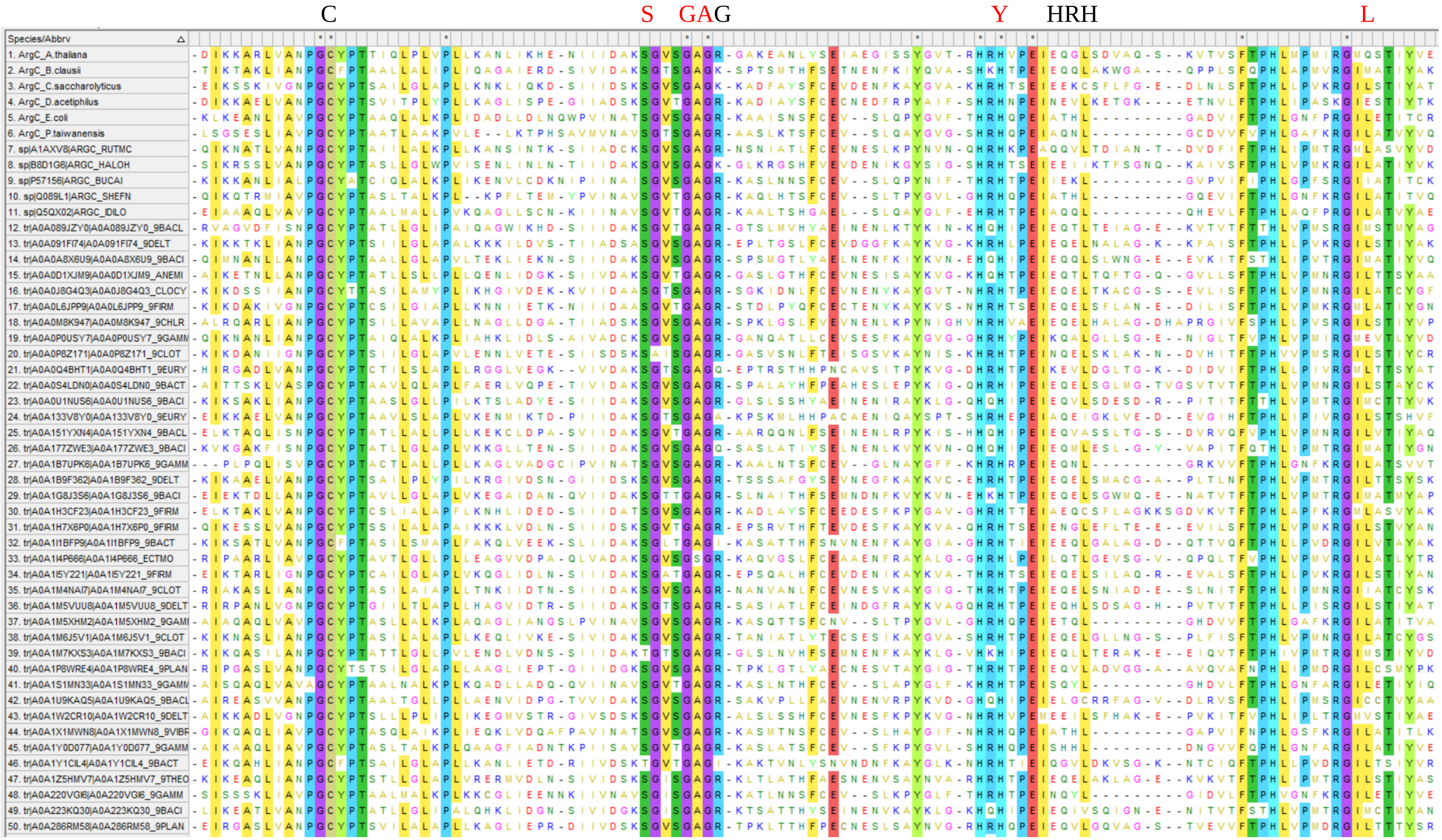

Supplementary Figure 8. Alignment of ArgC homologues. Conserved residues (> 70 %) are highlighted. Conserved active site cysteine, GAG motif and HRH motif are indicated. Target residues for saturation mutagenesis are labelled in red. Source data are provided as a Source Data file.

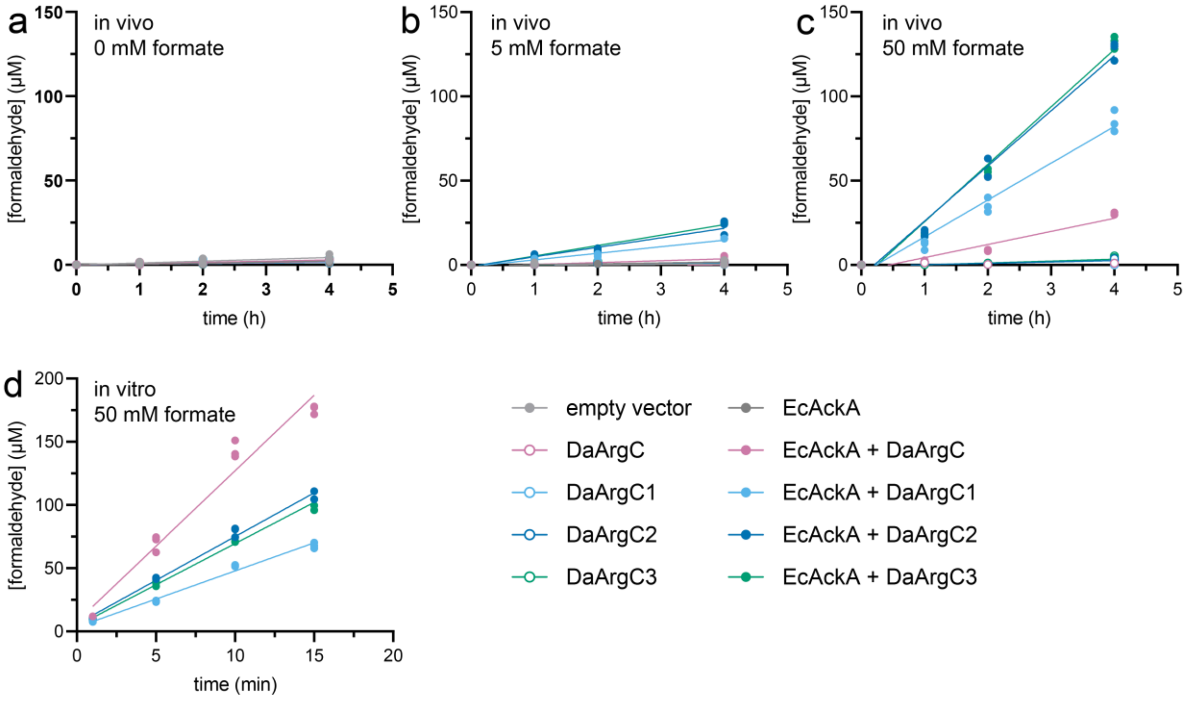

Supplementary Figure 9. Production plots of EcAckA+DaArgC with DaArgC variants. Formaldehyde concentration was determined by Nash assay. Shown are individual biological (**a-c**) or technical (**d**) replicates and linear fit (n=3). **a-c** *in vivo* activity on 0 mM, 5 mM, 50 mM ammonium formate. **d** activity of purified variants supplemented with 50 mM ammonium formate. EcAckA: *Escherichia coli* acetate kinase, DaArgC: *Denitrovibrio acetiphilus N*-acetyl-γ-glutamyl phosphate dehydrogenase. Source data are provided as a Source Data file.

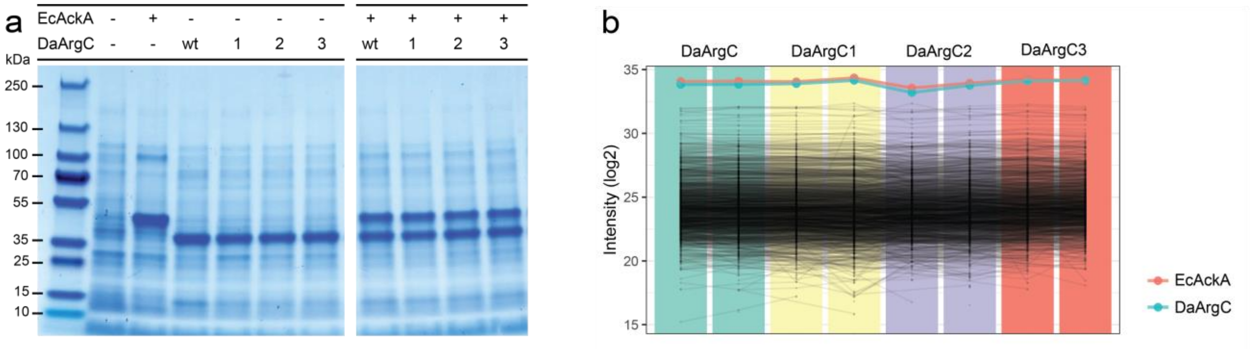

Supplementary Figure 10. ***In vivo*** **titer of DaArgC variants does not change with acquired mutations.** **a** SDS-Page of protein production in BL21DE3 Δ*frmRAB* Δ*pflAB*. Both sectors belong to the same gel. Two irrelevant bands were cropped out. m(EcAckA)=46 kDa, m(DaArgC)=38 kDa. The gel is a representative image for independent biological replicates (n=2). **b** Proteomic analysis of protein production in BL21DE3 Δ*frmRAB* Δ*pflAB*. Colored sectors indicate independent biological replicates of cultures expressing the same variant (n=2). Abundance for all shared peptides between the DaArgC variants is indicated as a colored line. Abundance of unrelated peptides are shown in black. EcAckA: *Escherichia coli* acetate kinase, DaArgC: *Denitrovibrio acetiphilus N*-acetyl-γ-glutamyl phosphate dehydrogenase. Source data are provided as a Source Data file.

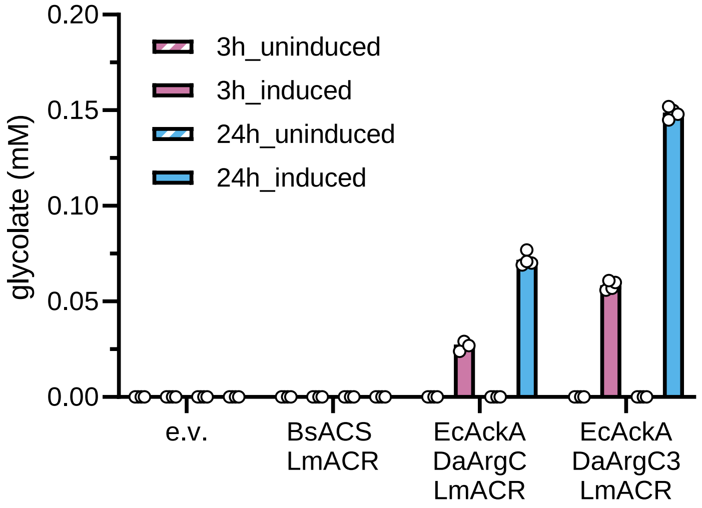

Supplementary Figure 11. Controls for resting cell bioconversion of formate to glycolate. Show is the mean and standard error of biological replicates (n=4) as well as individual measurements. BsACS: *Bacillus subtilis* acetyl-CoA synthase, EcAckA: *Escherichia coli* acetate kinase, DaArgC: *Denitrovibrio acetiphilus* *N*-acetyl-γ-glutamyl phosphate dehydrogenase, LmACR: *Listeria monocytogenes* acyl-CoA reductase. Source data are provided as a Source Data file.

a

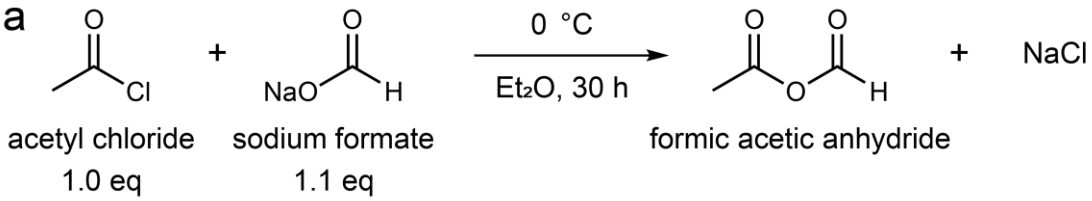

b

Compound	Integral	Molar ratio [%]
Formic acetic anh.	83.83	90.6
Acetic anh.	6.43 (/2)	3.5
Acetic acid	1.13	1.2
Diethyl ether	8.61(/2)	4.7

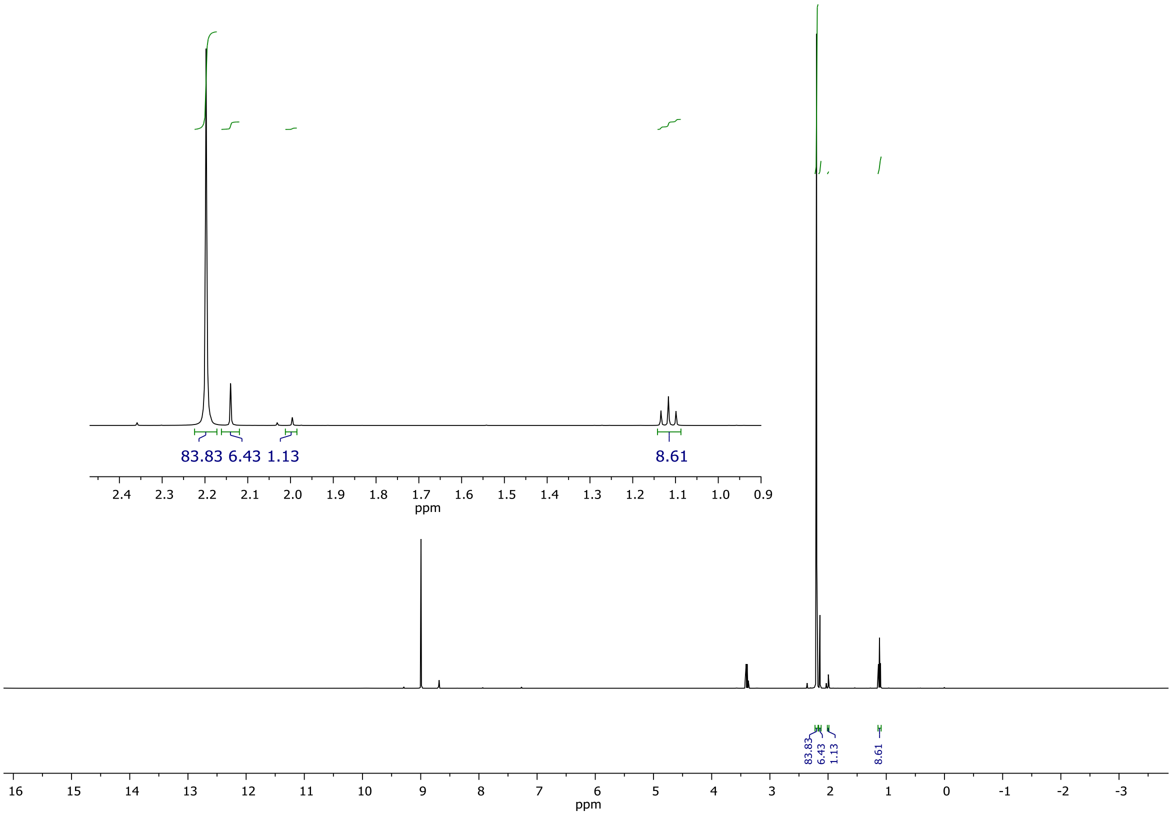

Supplementary Figure 12. Synthesis of formic acetic anhydride. a Reaction conditions for the synthesis of formic acetic anhydride. **b** ^{1}H-NMR of reaction mixture. Formic acetic anhydride shows a signal at 2.2 ppm for its CH_3 group and at 8.99 ppm for its CH group. Diethyl ether shows a triplet at 1.12 ppm and a quartet at 3.4 ppm. Side products are acetic acid at 2.00 ppm (CH_3), acetic anhydride at 2.14 ppm (CH_3) and residual signals exist for acetyl chloride (2.36 ppm) and sodium formate (8.68 ppm).

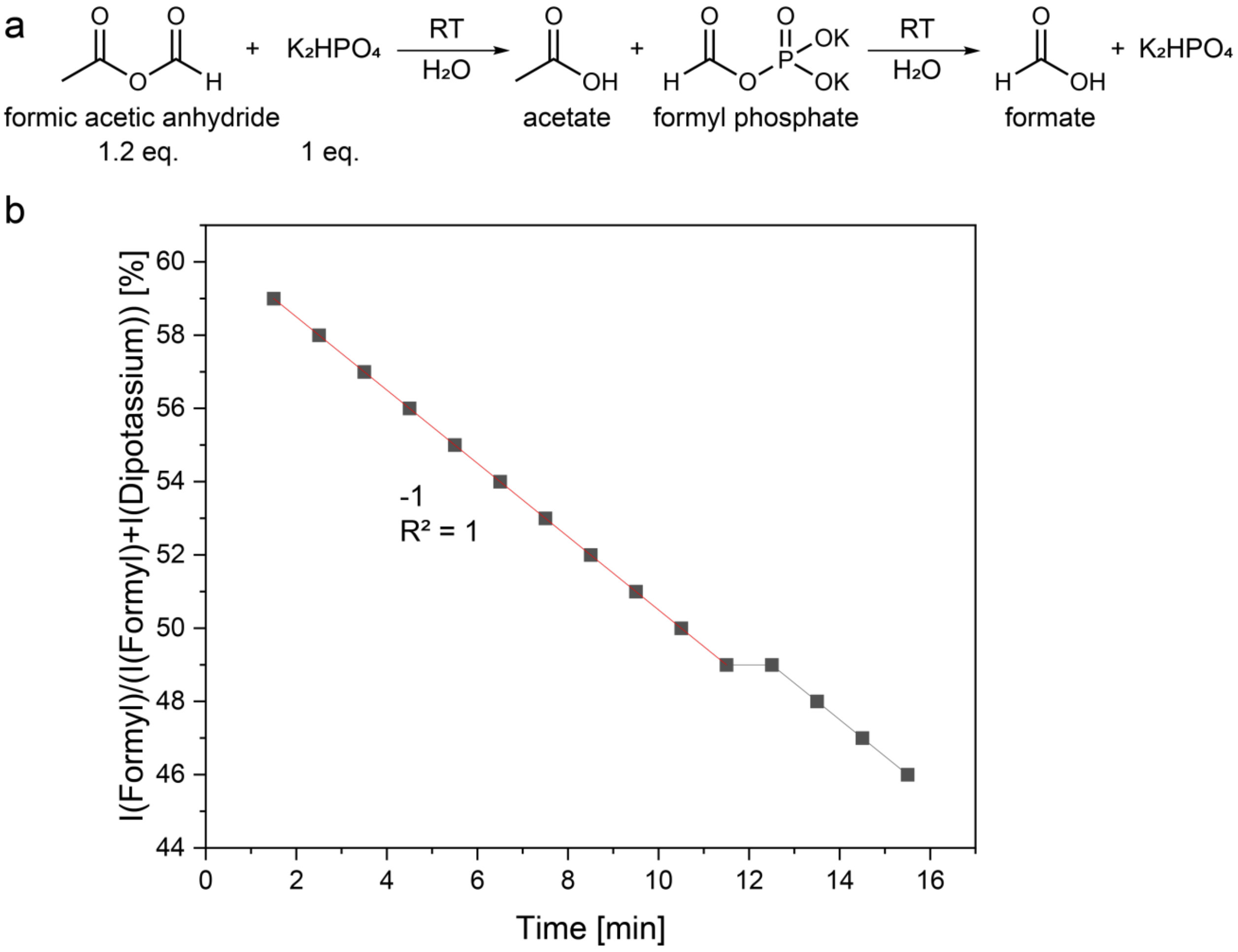

Supplementary Figure 13. Hydrolysis of formyl phosphate. a Scheme for the synthesis of formyl phosphate followed by its hydrolysis to formate and phosphate. **b** Hydrolysis rate of formyl phosphate as determined by quantitative $^{31}P\{^{1}H\}$-NMR integrals of formyl phosphate (formyl) and dipotassium phosphate (dipotassium). Only points on red line were included in linear fit. RT: room temperature. Source data are provided as a Source Data file.

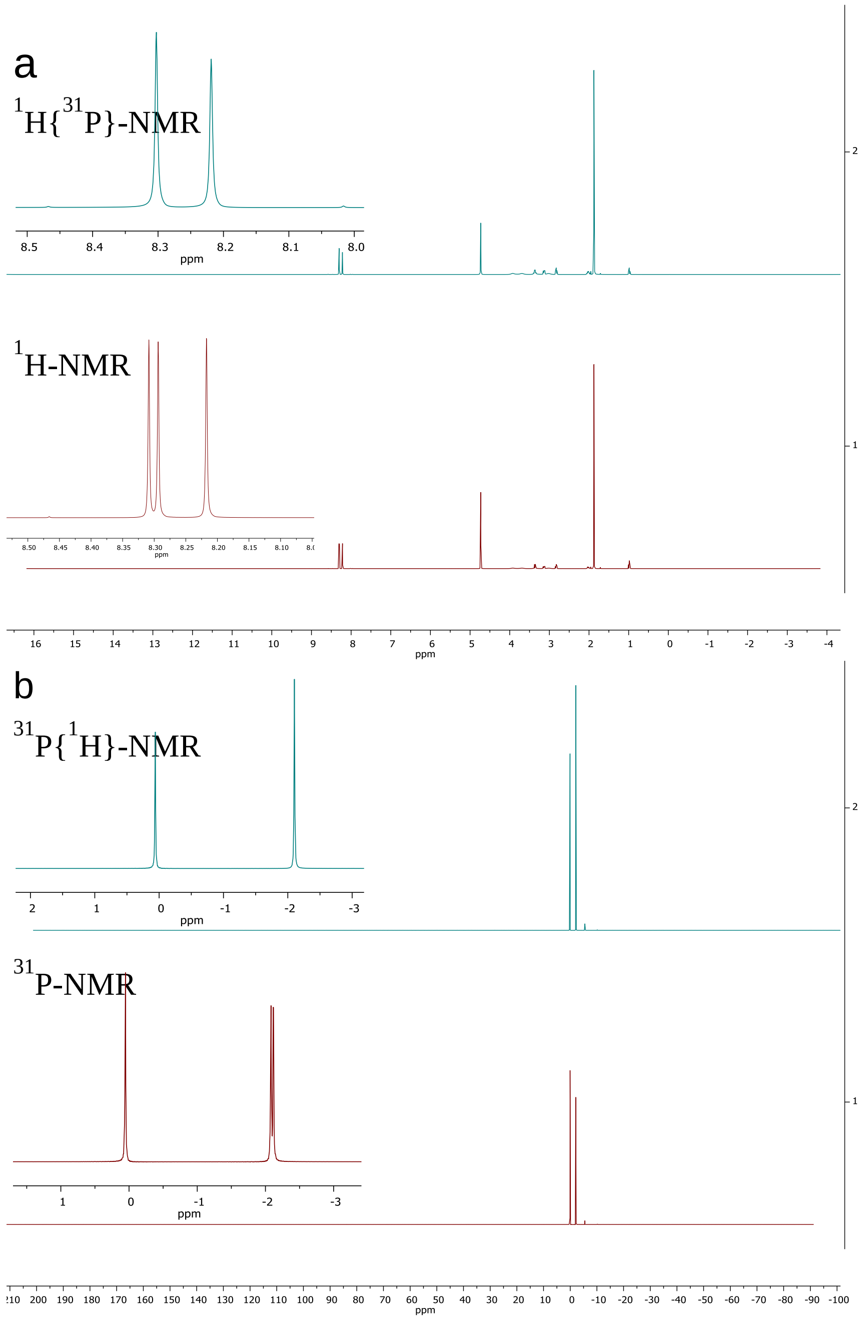

Supplementary Figure 14. Coupled and decoupled NMR of a mixture of formyl phosphate, formic acid and phosphate. Both the singlets at 8.3 ppm in ^{1}H-{^{31}P}-NMR and the one at -2.1 ppm in ^{31}P-{^{1}H}-NMR (**a** and **b** upper traces) become doublets with the same J_{PH}=5.8 Hz upon shutting off the decoupler (**a** and **b** bottom traces) allowing for a clear identification of the formyl phosphate signals. As expected, the singlet at 8.2 ppmin ^{1}H-NMR (formic acid) and the singlet at 0.1 ppm in ^{31}P-NMR (dipotassium phosphate) do not split when the decoupler is shut off.

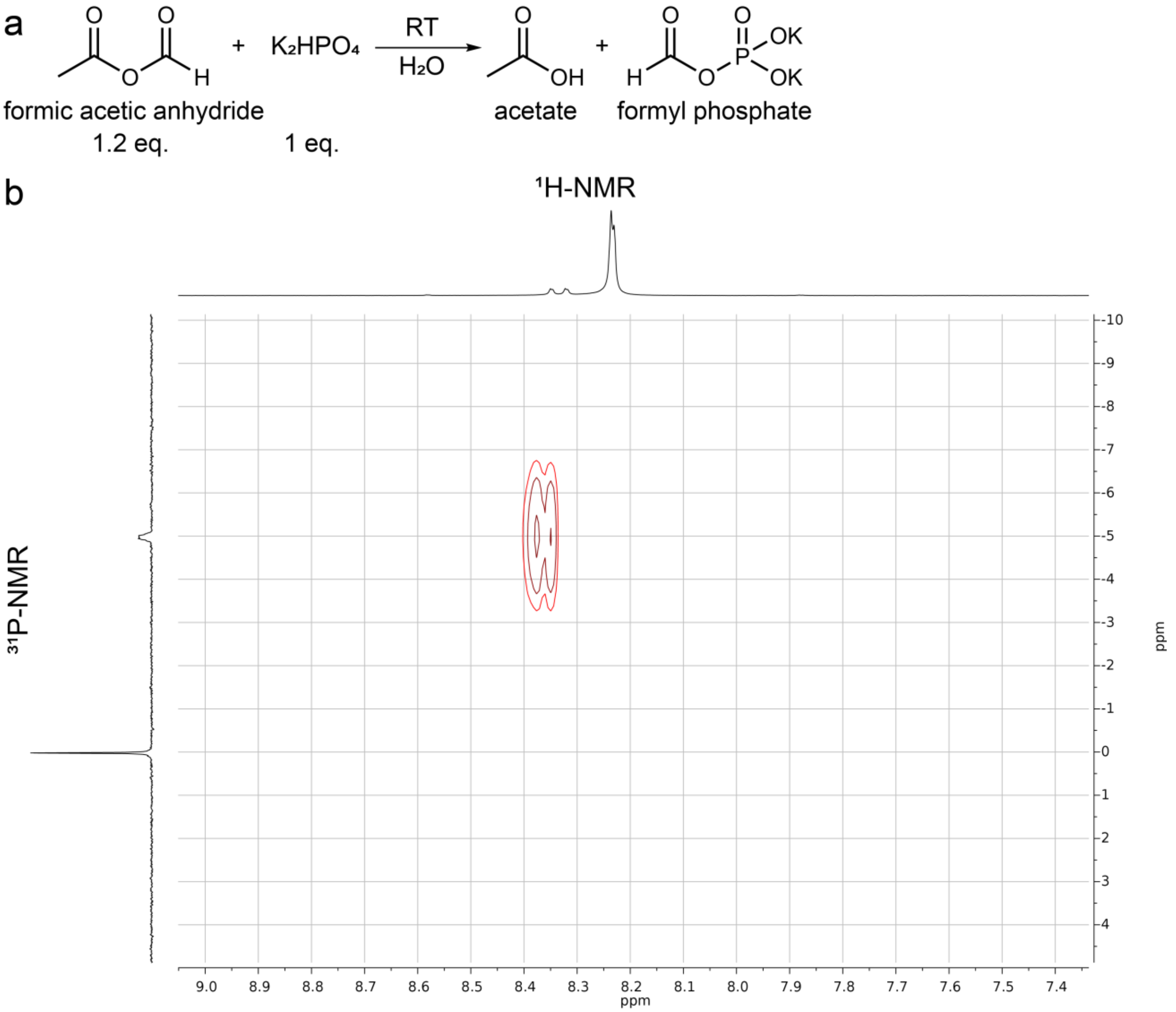

Supplementary Figure 15. ^{1}H-^{31}P-HMBC-NMR of formyl phosphate. a Reaction conditions for the synthesis of formyl phosphate. **b** ^{1}H-^{31}P-HMBC-NMR spectrum for formyl phosphate.

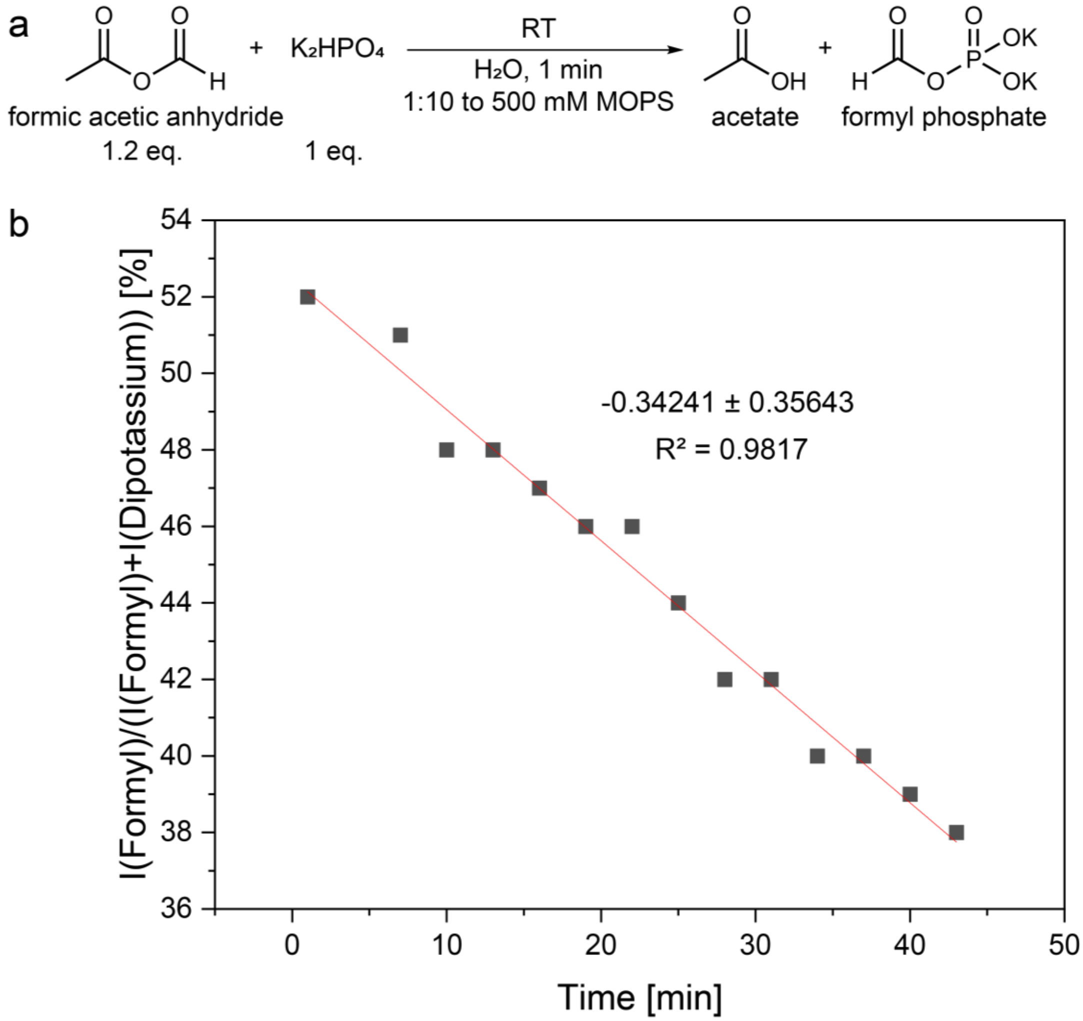

Supplementary Figure 16. Hydrolysis of formyl phosphate under reaction conditions. a Reaction conditions of formyl phosphate preparation for ArgC activity assays. **b** Hydrolysis rate of formyl phosphate as determined by quantitative $^{31}P\{^{1}H\}$-NMR integrals of formyl phosphate (formyl) and dipotassium. Source data are provided as a Source Data file.

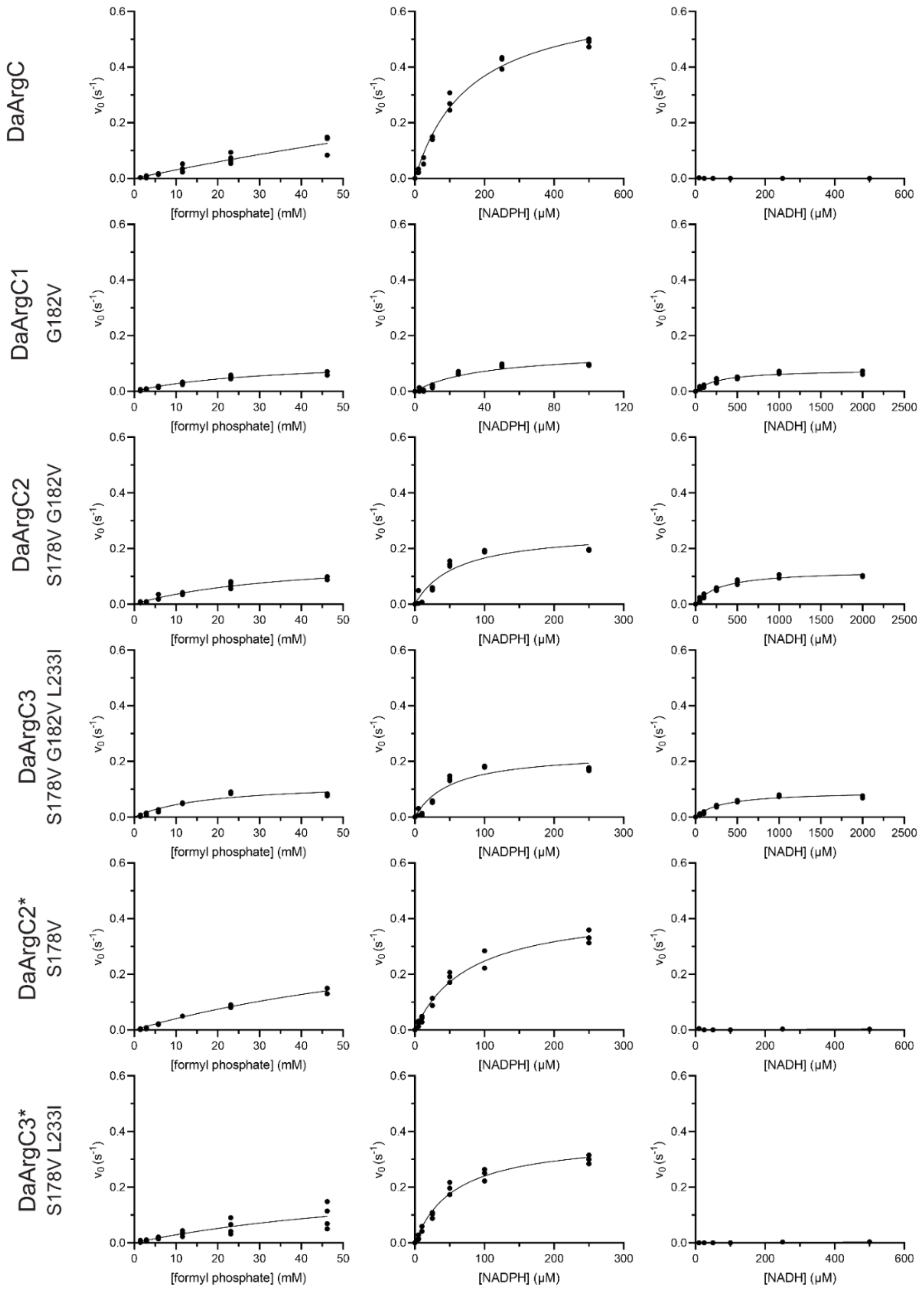

Supplementary Figure 17. Kinetic evaluation of DaArgC variants. Row 1: kinetics on formyl phosphate. Row 2: kinetics on NADPH, coupled to saturating concentrations of formate and non-limiting amounts of EcAckA. Row 3: kinetics on NADPH, coupled to saturating concentrations of formate and non-limiting amounts of EcAckA. Shown are individual technical replicates (n=4) and the Michaelis Menten fit. Source data are provided as a Source Data file.

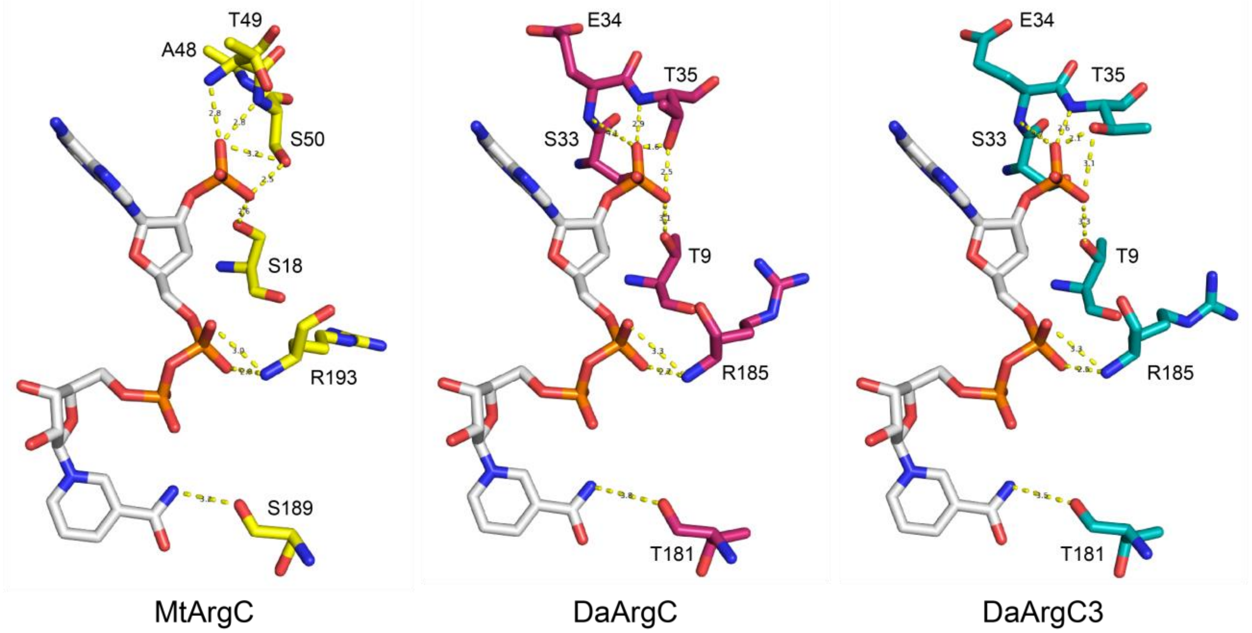

Supplementary Figure 18. NADPH coordination by ArgC variants. Cartoon depiction of the NADPH-binding domains of MtArgC (PBD-ID 2I3G [http://doi.org/10.2210/pdb2i3g/pdb]) and the putative NADPH-binding domains of DaArgC (PBD-ID 8AFU [https://doi.org/10.2210/pdb8AFU/pdb]) and DaArgC3 (PBD-ID 8AFV [https://doi.org/10.2210/pdb8AFV/pdb]). Relevant residues are labelled. DaArgC: *Denitrovibrio acetiphilus* *N*-acetyl-γ-glutamyl phosphate dehydrogenase, MtArgC: *Mycobacterium tuberculosis* *N*-acetyl-γ-glutamyl phosphate dehydrogenase

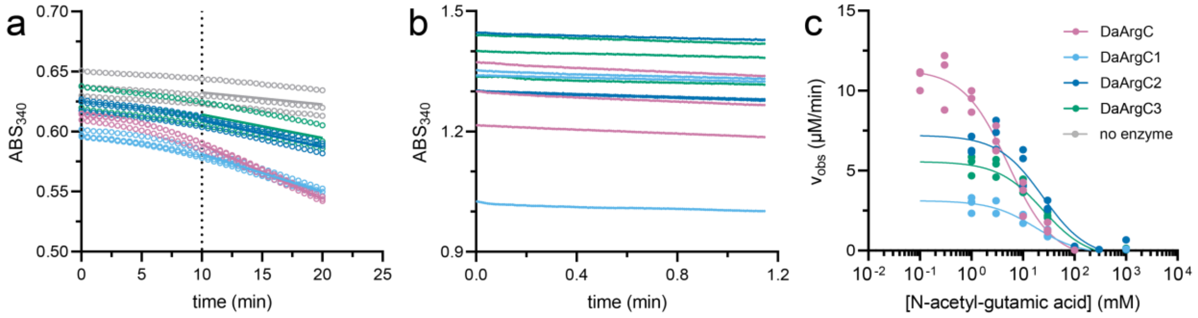

Supplementary Figure 19. Activity of DaArgC variants on acetyl phosphate and *N*-acetyl-γ-glutamyl phosphate, and inhibition of P_i route by *N*-acetyl-γ-glutamic acid. a DaArgC activity on acetyl phosphate. Shown are individual technical replicates (n=3) and linear fit. Dotted line indicates the time point from which the linear fit was applied. **b** DaArgC activity on *N*-acetyl-γ-glutamyl phosphate. Shown are individual technical replicates (n=3). **c** Inhibition of DaArgC variants by *N*-acetyl-γ-glutamic acid. Shown are inhibitor response least squares fit and individual technical replicates (n=3). *Denitrovibrio acetiphilus* *N*-acetyl-γ-glutamyl phosphate dehydrogenase. Source data are provided as a Source Data file.

>sp|P0A6A3|ACKA_ECOLI Acetate kinase
OS=Escherichia coli (strain K12) GN=ackA PE=1 SV=1 EcAckA
MGSSHHHHHHSQDPALRASSKLVLVLNCGSSSLKFAIIDAVNGEEYLSGLAECFHLPEAR
IKWKMDGNKQEAALGAGAAHSEALNFIVNTILAQKPELSAQLTAIGHRIVHGGEKYTSSV
VIDESVIQGIKDAASFAPLHNPAHLIGIEEALKSFPQLKDKNVAVFDTAFHQTMPEESYL
YALPYNLYKEHGIRRYGAHGTSHFYVTQEAAKMLNKPVEELNIITCHLGNGGSVSAIRNG
KCVDTSMGLTPLEGLVMGTRSGDIDPAIIFHLHDTLGMSVDAINKLLTKESGLLGLTEVT
SDCRYVEDNYATKEDAKRAMDVYCHRLAKYIGAYTALMDGRLDAVVFTGGIGENAAMVRE
LSLGKLGVLGFEVDHERNLAARFGKSGFINKEGTRPAVVIPTNEELVIAQDASRLTAGLC
GR

>sp|X00001|DaArgC DaArgC OS=Custom GN=DaArgC PE=1 SV=1 DaArgC
MMKVSVIGATGYTGYELVKILANHPEFEIAALVSETYADKMFSDVYPRLRSICDVVITGR
DYDAVAEISDAVFLCLPHAAAQDAAAFFYEKGLKVVDFSADFRLKDKKLYEATYKVDHTY
PDLLRKAVYGLPEIFEVDIKKAELVANPGCYPTSVITPLYPLLKADIAYSFCECNEDFRP
YAIFSHRHNPEINEVLKGIESTIYTKTTAGLAEISACLKDFYRERRCVRIYDNGHIPSTA
DVTDTNFIDIGLFVKGERLIIVSCIDNLIKGSSGMAVQNMNLMCGFDDTLGIL

The original DaArgC sequence is shown below. Sequences removed for analysis are marked in red:

>DaArgC
MMKVSVIGATGYTGYELVKILANHPEFEIAALVSETYADKMFSDVYPRLRSICDVVITGR
DYDAVAEISDAVFLCLPHAAAQDAAAFFYEKGLKVVDFSADFRLKDKKLYEATYKVDHTY
PDLLRKAVYGLPEIFEVDIKKAELVANPGCYPTSVITPLYPLLKAGLISPEGIIADSKSG
VTGAGRKADIAYSFCECNEDFRPYAIFSHRHNPEINEVLKETGKETNVLFTPHLIPASKG
IESTIYTKTTAGLAEISACLKDFYRERRCVRIYDNGHIPSTADVTDTNFIDIGLFVKGER
LIIVSCIDNLIKGSSGMAVQNMNLMCGFDDTLGIL

Removed sequences coming from different isoforms are marked in red below:

>DaArgC wild type
AGLISPEGIIADSKSGVTGAGRK...ETGKETNVLFTPHLIPASK

>DaArgC1 [G182V]
AGLISPEGIIADSKSGVTVAGRK...ETGKETNVLFTPHLIPASK

>DaArgC2 [S178V;G182V]
AGLISPEGIIADSKVGVTVAGRK...ETGKETNVLFTPHLIPASK

>DaArgC3 [S178V;G182V;L233I]
AGLISPEGIIADSKVGVTVAGRK...ETGKETNVLFTPHIIPASK

Supplementary Figure 20. Sequences used in proteomic analysis.

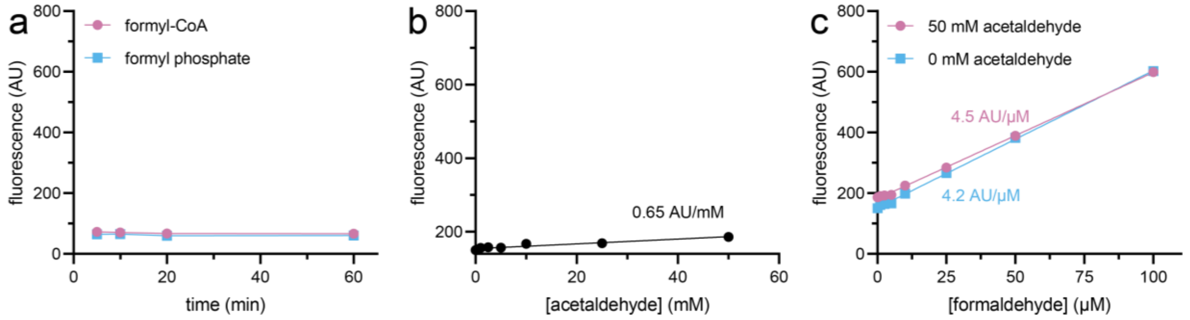

Supplementary Figure 21. Controls for the Nash reaction. a reactivity of Nash reagent with formyl-CoA and formyl phosphate. Formyl-CoA and formyl phosphate were produced *in situ* by FCS and FOK, respectively. **b** reactivity of Nash reagent with acetaldehyde. **c** Reactivity of Nash reagent with formaldehyde in the presence and absence of 50 mM acetaldehyde. For all panels, the linear fit of single measurements (n=1) is shown. AU: arbitrary units. Source data are provided as a Source Data file.

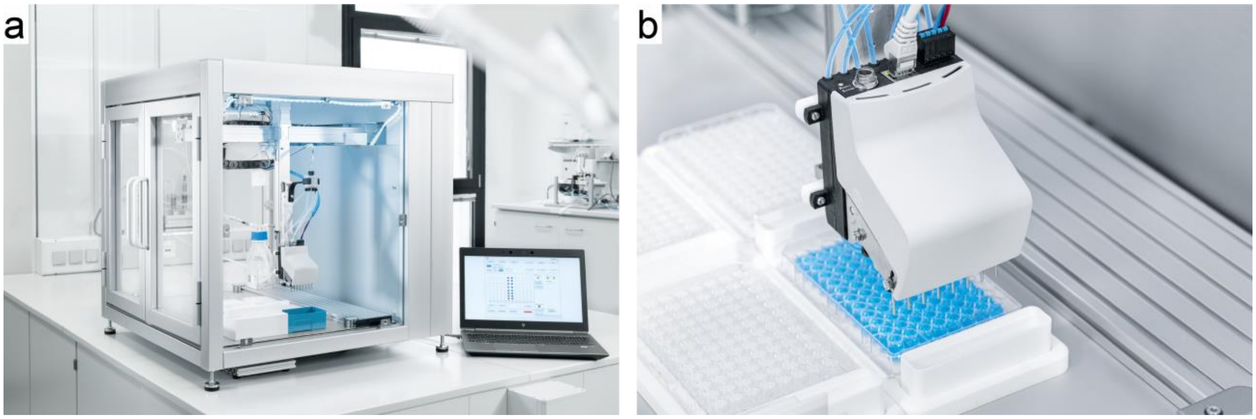

Supplementary Figure 22. Images of the liquid handler. a view of the entire setup. **b** close-up of the dispensing head and interior setup. Images were adapted from Festo SE & Co. KG (2022).

Supplementary Table 1. Energy profile of formate reduction routes by eQuilibrator.

Reaction formula	$\Delta_r G°$ (kJ/mol)	$\Delta_r G'^m$ (kJ/mol)	Route
formate+ATP+THF=formyl-THF+ADP+Pi	-2.67	-5.88	THF
formyl-THF+NADPH=methylene-THF+NADP$^+$+H_2O	-4.07	-4.84	THF
formyl-THF+NADH=methylene-THF+NAD$^+$+H_2O	-4.21	-4.98	THF
methylene-THF+H_2O=formaldehyde+THF	25.36	6.86	THF
formate+ATP+CoA=formyl-CoA+AMP+PPi	-30.95	-17.60	CoA
formyl-CoA+NADPH=formaldehyde+CoA+NADP$^+$	39.41	8.09	CoA
formyl-CoA+NADH=formaldehyde+CoA+NAD$^+$	39.28	7.95	CoA
formate+ATP=formyl-phosphate+ADP	-15.43	-9.79	Pi
formyl-phosphate+NADPH=formaldehyde+NADP$^+$+Pi	34.04	7.32	Pi
formyl-phosphate+NADH=formaldehyde+NAD$^+$+Pi	33.91	2.78	Pi

$\Delta_r G°$: estimated change in Gibbs free energy in standard conditions; $\Delta_r G'^m$: estimated change in Gibbs free energy in conditions pH 7.0, pMg 3.0 and ionic strength 0.25 mM, with all compounds at 1 mM concentration.

Supplementary Table 2. Literature efficiencies of ACS and ACR variants. Where available, standard deviation is indicated.

Enzyme	K_m (mM)	k_{cat} (s^{-1})	Productivity (µM formaldehyde/s)	Publication
StACS	~ 120	~ 12		[2]
MhACS	~ 90	~ 6		[2]
EcACS	~ 50	~ 8		[2]
ArACS	~ 40	~ 6		[2]
TtACS	~ 150	~ 5		[2]
SdACS	~ 150	~ 9		[2]
LmACR	10.4 ± 2.2	0.99 ± 0.07		[3]
ACS-BmACR			~ 0.14 (lysate)	[2]
ACS-LmACR			~ 0.2 (lysate)	[2]
BsACS-LmACR			~ 0.08 (*in vitro*)	[4]
BsACS-LmACR			~ 0.08 (*in vitro*)	[4]

Supplementary Table 3. Kinetic parameters of variants DaArgC2* and DaArgC3*.

Enzyme	Formyl phosphate			NADPH [(1)]			NADH[(1)]		
	$K_{m, app}$ (mM)	$k_{cat, app}$ (s^{-1})	k_{cat}/K_m ($M^{-1}s^{-1}$)	$K_{m, app}$ (mM)	$k_{cat, app}$ (s^{-1})	k_{cat}/K_m ($M^{-1}s^{-1}$)	$K_{m, app}$ (mM)	$k_{cat, app}$ (s^{-1})	k_{cat}/K_m ($M^{-1}s^{-1}$)
DaArgC2*	no fit	0.14 ± 0.01[(2)]	-	0.08 ± 0.01	0.44 ± 0.02	$5.5*10^3$	>500[(3).]	<0.005[(3).]	<1[(3)]
DaArgC3*	no fit	0.10 ± 0.05[(2)]	-	0.06 ± 0.01	0.38 ± 0.02	$6.3*10^3$	>500[(3).]	<0.005[(3).]	<1[(3)]

Parameters were determined by Michaelis Menten fit (see Supplementary Fig. 15). Standard error of independent technical triplicates (n=3) is indicated. [(1)] indicates measurements coupled to EcAckA. [(2)] maximal activity observed, [formyl-phosphate]=46 mM. [(3)] activity below detection limit.

Supplementary Table 4. Bacterial strains.

Strain	Genotype	Use	Source
E. coli BL21 (DE3)	*E. coli str. F⁻ ompT gal dcm lon* $hsdS_B(r_B^- m_B^-)$ *λ(DE3 [lacI lacUV5-T7p07 ind1 sam7 nin5]) [malB⁺]*$_{K-12}(\lambda^S)$	protein production	Invitrogen
E. coli BL21 (DE3) Δ*frmRAB*	*E. coli* BL21 (DE3) Δ*frmRAB::Kan*	protein production	Ari Satanowski, MPI Marburg
E. coli BL21 (DE3) Δ*frmRAB* Δ*pflAB*	*E. coli* BL21 (DE3) Δ*frmRAB* Δ*pflAB*	protein production	This study
E. coli DH5α	*E. coli* F⁻ *φ*80*lac*ZΔM15 Δ(*lac*ZYA-*arg*F)U169 *rec*A1 *end*A1 *hsd*R17(r_K^-, m_K^+) *pho*A *sup*E44 λ⁻*thi*-1 *gyr*A96 *rel*A1	vector propagation, cloning	Invitrogen
E. coli AC440	*E. coli* MG1655(DE3) Δ*frmA* Δ*fdhF* Δ*fdnG* Δ*fdoG* Δ*glcD*::FRT	resting cell bioconversion	[3]
E. coli AC440 Δ*patZ*	*E. coli* MG1655(DE3) Δ*frmA* Δ*fdhF* Δ*fdnG* Δ*fdoG* Δ*glcD*::FRT ΔpatZ	resting cell bioconversion (for BsACS)	

Supplementary Table 5. Data collection and refinement statistics. Values in parentheses are for highest-resolution shell.

	DaArgC	DaArgC3
Data collection		
Beam line	ESRF ID30B	ESRF ID30B
Wavelength (Å)	0.97625	0.97625
Space group	I 2	$P\ 2_1\ 2_1\ 2_1$
Cell dimensions		
a, *b*, *c* (Å)	86.7, 77.8, 122.0	91.9, 109.6, 133.2
α, β, γ (°)	90.0, 92.9, 90.0	90.0, 90.0, 90.0
Unique reflections	55250 (5200)	66892 (6707)
Resolution (Å)	42.69 - 1.99 (2.06 - 1.99)	42.31 - 2.194 (2.27 - 2.19)
R_{merge}	0.02854 (0.2347)	0.03863 (0.1318)
$I/\sigma(I)$	13.45 (2.91)	13.50 (5.56)
$CC_{1/2}$	0.999 (0.936)	0.996 (0.952)
Completeness (%)	99.13 (93.33)	96.33 (97.87)
Redundancy	6.5 (6.5)	3.2 (3.2)
Refinement		
No. reflections	55208 (5191)	66884 (6707)
R_{work}/R_{free}	0.1843/0.2152	0.1883/0.2069
No. atoms (non-hydrogen)	5439	10561
Protein	5217	10023
Ligand/ion	2 Na^+	4 Na^+
Water	220	534
B-factors (average)	45.76	38.56
Protein	45.62	38.44
Ligand/ion	35.60	32.98
Water	49.11	40.84
R.m.s. deviations		
Bond lengths (Å)	0.014	0.004
Bond angles (°)	1.220	0.680
Rotamer outliers (%)	0.35	0.37
Ramachandran favored (%)	97.45	96.26
Ramachandran outliers (%)	0.30	0.31
PDB ID	8AFU	8AFV

Supplementary Table 6. Parameter settings for LC-MS detection of glycolyl-CoA.

Compound	Quantifier	Collision energy	Qualifier	Collision energy	Dwell	Fragmenter voltage	Cell accelerater volatege
Glycolyl-CoA	826.1→319.4	24	826.1→428.4	25	200	140	5

Supplementary Table 7. Bacterial strains for resting cell bioconversion.

Route	Strain	Plasmids
EcAckA+DaArgC+LmACR+BsmHACL+EcAldA	*E. coli* AC440	pCDFDuet-1_BsmHACL_DaArgC<br>pETDuet-1_P^{CT5}_lmACR-ecAckA-ecAldA
EcAckA+DaArgC3+LmACR+BsmHACL+EcAldA	*E. coli* AC440	pCDFDuet-1_BsmHACL_DaArgC3<br>pETDuet-1_P^{CT5}_LmACR-EcAckA-EcAldA
BsACS+LmACR+BsmHACL+EcAldA	*E. coli* AC440 Δ*patZ*	pCDFDuet-1_BsmHACL_BsACS<br>pETDuet-1_P^{CT5}_LmACR-EcAldA

Supplementary references

[1] Krimen, L. I. Acetic formic anhydride. *Org. Synth.* **50**, 1-2 (1970).

[2] Wang, J., Anderson, K., Yang, E., He, L. & Lidstrom, M. E. Enzyme engineering and *in vivo* testing of a formate reduction pathway. *Synth. Biol.* **6**, ysab020 (2021).

[3] Chou, A., Clomburg, J. M., Qian, S. & Gonzalez, R. 2-Hydroxyacyl-CoA lyase catalyzes acyloin condensation for one-carbon bioconversion. *Nat. Chem. Biol.* **15**, 900-906 (2019).

[4] Hu, G. *et al.* Synergistic metabolism of glucose and formateincreases the yield of short-chain organic acids in *Escherichia coli*. *ACS Synth. Biol.* **11**, 135-143 (2022).
