# Supplementary material for: Engineering a new-to-nature cascade for phosphate-dependent formate to formaldehyde conversion in vitro and in vivo
Source: Nat Commun. 2023 May 9;14:2682. doi: 10.1038/s41467-023-38072-w (PMC10170137; doi:10.1038/s41467-023-38072-w)
Supplement: Supplementary file 2 — Description of Additional Supplementary Files [file 41467_2023_38072_MOESM2_ESM.pdf]

Description of Additional Supplementary Files

File Name: Supplementary Data 1
Description: Reagents.

File Name: Supplementary Data 2
Description: Plasmids.

File Name: Supplementary Data 3
Description: Primers.

File Name: Supplementary Data 4
Description: Golden Gate construction.
