# Supplementary material for: Engineering a new-to-nature cascade for phosphate-dependent formate to formaldehyde conversion in vitro and in vivo
Source: Nat Commun. 2023 May 9;14:2682. doi: 10.1038/s41467-023-38072-w (PMC10170137; doi:10.1038/s41467-023-38072-w)
Supplement: Supplementary file 7 — Reporting Summary [file 41467_2023_38072_MOESM7_ESM.pdf]

nature portfolio

Corresponding author(s): Tobias J. Erb

Last updated by author(s): Mar 29, 2023

Reporting Summary

Nature Portfolio wishes to improve the reproducibility of the work that we publish. This form provides structure for consistency and transparency in reporting. For further information on Nature Portfolio policies, see our Editorial Policies and the Editorial Policy Checklist.

Statistics

For all statistical analyses, confirm that the following items are present in the figure legend, table legend, main text, or Methods section.

n/a	Confirmed	
☐	☒	The exact sample size (*n*) for each experimental group/condition, given as a discrete number and unit of measurement
☐	☒	A statement on whether measurements were taken from distinct samples or whether the same sample was measured repeatedly
☒	☐	The statistical test(s) used AND whether they are one- or two-sided<br>*Only common tests should be described solely by name; describe more complex techniques in the Methods section.*
☒	☐	A description of all covariates tested
☐	☒	A description of any assumptions or corrections, such as tests of normality and adjustment for multiple comparisons
☐	☒	A full description of the statistical parameters including central tendency (e.g. means) or other basic estimates (e.g. regression coefficient) AND variation (e.g. standard deviation) or associated estimates of uncertainty (e.g. confidence intervals)
☒	☐	For null hypothesis testing, the test statistic (e.g. *F*, *t*, *r*) with confidence intervals, effect sizes, degrees of freedom and *P* value noted<br>*Give P values as exact values whenever suitable.*
☒	☐	For Bayesian analysis, information on the choice of priors and Markov chain Monte Carlo settings
☒	☐	For hierarchical and complex designs, identification of the appropriate level for tests and full reporting of outcomes
☒	☐	Estimates of effect sizes (e.g. Cohen's *d*, Pearson's *r*), indicating how they were calculated

Our web collection on statistics for biologists contains articles on many of the points above.

Software and code

Policy information about availability of computer code

Data collection	Tecan iControl v3.8.2.0, Cary WinUV Kinetics application v5.0.0.999 , Agilent MassHunter Quantitative Analysis (QQQ) v10.0, BLAST v2.13.0, MxCube3 v2.3.0.1
Data analysis	GraphPad Prism v9.0.2, Agilent MassHunter, Cary WinUV Kinetics application v5.0.0.999 , Microsoft Excel v2208, HH-suite version 3, MEGA X v10.0.5 , XDS Built=20200417, CCP4 program suite v8.0.004, Phenix, Coot, python packages equilibrator_api v 0.4.7 and equilibrator_pathway v 0.4.7, COBRApy (v 0.20.0), DIA-NN, Phenix v1.18.2-3874, XDS Built=20200417, SCALA v3.3.22, Phenix v1.18.2-3874 , Phaser v2.8.3, custom code (https://github.com/he-hai/PubSuppl)

For manuscripts utilizing custom algorithms or software that are central to the research but not yet described in published literature, software must be made available to editors and reviewers. We strongly encourage code deposition in a community repository (e.g. GitHub). See the Nature Portfolio guidelines for submitting code & software for further information.

Data

Policy information about availability of data

All manuscripts must include a data availability statement. This statement should provide the following information, where applicable:

- Accession codes, unique identifiers, or web links for publicly available datasets
- A description of any restrictions on data availability
- For clinical datasets or third party data, please ensure that the statement adheres to our policy

Source data is provided with this paper. Raw data of plate reader and CARY experiments is supplied in the Source Data file. Raw data of LC-MS measurements are

deposited in Edmond [https://doi.org/10.17617/3.BKLI0C]. X-Ray structures can be accessed on PDB under accession codes 8AFU [https://doi.org/10.2210/pdb8AFU/pdb] (DaArgC) and 8AFV [https://doi.org/10.2210/pdb8AFV/pdb] (DaArgC3). Proteomic data is available on ProteomeXchange under accession code PXD041037 [http://proteomecentral.proteomexchange.org/cgi/GetDataset?ID=PXD041037] and on MassIVE [ftp://massive.ucsd.edu/MSV000091532/].

Human research participants

Policy information about studies involving human research participants and Sex and Gender in Research.

Reporting on sex and gender	There were no human research participants in this study.
Population characteristics	See above.
Recruitment	See above.
Ethics oversight	See above.

Note that full information on the approval of the study protocol must also be provided in the manuscript.

Field-specific reporting

Please select the one below that is the best fit for your research. If you are not sure, read the appropriate sections before making your selection.

[x] Life sciences   [ ] Behavioural & social sciences   [ ] Ecological, evolutionary & environmental sciences

For a reference copy of the document with all sections, see nature.com/documents/nr-reporting-summary-flat.pdf

Life sciences study design

All studies must disclose on these points even when the disclosure is negative.

Sample size	No sample size determination was performed. Sample size ranged from 1 to 4 depending on experiment. Unicates were only applied in method establishment and not used to draw wider conclusions. Duplicates were used in the analysis of protein titer in the cells and surface to provide a trend for protein production. All biochemical experiments were performed in sample sizes of 3 or 4, which are sufficient to provide reasonable confidence in their results.
Data exclusions	Data was excluded when clear outliers were observed. Criteria for data exclusion where not pre-established.
Replication	Datasets shown in this study were reproducible in a minimum of two independent technological/ biological replicates performed on different days using the same conditions.
Randomization	There was no allocation of samples into experimental groups as randomization did not benefit measurement or evaluation of kinetic data.
Blinding	Blinding of the study was not possible as experiments were performed by a single researcher, who knew the setup of all samples.

Reporting for specific materials, systems and methods

We require information from authors about some types of materials, experimental systems and methods used in many studies. Here, indicate whether each material, system or method listed is relevant to your study. If you are not sure if a list item applies to your research, read the appropriate section before selecting a response.

Materials & experimental systems

n/a	Involved in the study	
[x]	[ ]	Antibodies
[x]	[ ]	Eukaryotic cell lines
[x]	[ ]	Palaeontology and archaeology
[x]	[ ]	Animals and other organisms
[x]	[ ]	Clinical data
[x]	[ ]	Dual use research of concern

Methods

n/a	Involved in the study	
[x]	[ ]	ChIP-seq
[x]	[ ]	Flow cytometry
[x]	[ ]	MRI-based neuroimaging
